# Comparative and functional genomics provide insights into the pathogenicity of dermatophytic fungi

**DOI:** 10.1186/gb-2011-12-1-r7

**Published:** 2011-01-19

**Authors:** Anke Burmester, Ekaterina Shelest, Gernot Glöckner, Christoph Heddergott, Susann Schindler, Peter Staib, Andrew Heidel, Marius Felder, Andreas Petzold, Karol Szafranski, Marc Feuermann, Ivo Pedruzzi, Steffen Priebe, Marco Groth, Robert Winkler, Wenjun Li, Olaf Kniemeyer, Volker Schroeckh, Christian Hertweck, Bernhard Hube, Theodore C White, Matthias Platzer, Reinhard Guthke, Joseph Heitman, Johannes Wöstemeyer, Peter F Zipfel, Michel Monod, Axel A Brakhage

**Affiliations:** 1 Department of Molecular and Applied Microbiology, Leibniz Institute for Natural Product Research and Infection Biology - Hans Knöll Institute (HKI), Beutenbergstrasse 11a, Jena, 07745, Germany; 2 Institute of Microbiology, Friedrich Schiller University (FSU) Jena, Neugasse 24, Jena, 07743, Germany; 3 Systems Biology/Bioinformatics group, Leibniz Institute for Natural Product Research and Infection Biology - Hans Knöll Institute (HKI), Beutenbergstrasse 11a, Jena, 07745, Germany; 4 Genome Analysis group, Leibniz Institute for Age Research - Fritz Lipmann Institute (FLI), Beutenbergstrasse 11, Jena, 07745, Germany; 5 Department of Infection Biology, Leibniz Institute for Natural Product Research and Infection Biology - Hans Knöll Institute (HKI), Beutenbergstrasse 11a, Jena, 07745, Germany; 6 Friedrich Schiller University (FSU) Jena, Fürstengraben 26, Jena, 07743, Germany; 7 Junior Research Group Fundamental Molecular Biology of Pathogenic Fungi, Leibniz Institute for Natural Product Research and Infection Biology - Hans Knöll Institute (HKI), Beutenbergstrasse 11a, Jena, 07745, Germany; 8 Biocomputing group, Leibniz Institute for Age Research - Fritz Lipmann Institute (FLI), Beutenbergstrasse 11, Jena, 07745, Germany; 9 Swiss-Prot group, SIB, Swiss Institute of Bioinformatics, 1 rue Michel Servet, Geneve, 1204, Switzerland; 10 Department of Biomolecular Chemistry, Leibniz Institute for Natural Product Research and Infection Biology - Hans Knöll Institute (HKI), Beutenbergstrasse 11a, Jena, 07745, Germany; 11 Department of Molecular Genetics and Microbiology, Duke University Medical Center, 322 CARL Building, Box 3546 DUMC, Durham, NC 27710, USA; 12 Department of Microbial Pathogenicity Mechanisms, Leibniz Institute for Natural Product Research and Infection Biology - Hans Knöll Institute (HKI), Beutenbergstrasse 11a, Jena, 07745, Germany; 13 Seattle Biomedical Research Institute, University of Washington, 307 Westlake Ave, N., Suite 500, Seattle, WA 98109-5219, USA; 14 Department of Dermatology, Centre Hospitalier Universitaire Vaudois, Lausanne, CH-1011, Switzerland

## Abstract

**Background:**

Millions of humans and animals suffer from superficial infections caused by a group of highly specialized filamentous fungi, the dermatophytes, which exclusively infect keratinized host structures. To provide broad insights into the molecular basis of the pathogenicity-associated traits, we report the first genome sequences of two closely phylogenetically related dermatophytes, *Arthroderma benhamiae *and *Trichophyton verrucosum*, both of which induce highly inflammatory infections in humans.

**Results:**

97% of the 22.5 megabase genome sequences of *A. benhamiae *and *T. verrucosum *are unambiguously alignable and collinear. To unravel dermatophyte-specific virulence-associated traits, we compared sets of potentially pathogenicity-associated proteins, such as secreted proteases and enzymes involved in secondary metabolite production, with those of closely related onygenales (*Coccidioides *species) and the mould *Aspergillus fumigatus*. The comparisons revealed expansion of several gene families in dermatophytes and disclosed the peculiarities of the dermatophyte secondary metabolite gene sets. Secretion of proteases and other hydrolytic enzymes by *A. benhamiae *was proven experimentally by a global secretome analysis during keratin degradation. Molecular insights into the interaction of *A. benhamiae *with human keratinocytes were obtained for the first time by global transcriptome profiling. Given that *A. benhamiae *is able to undergo mating, a detailed comparison of the genomes further unraveled the genetic basis of sexual reproduction in this species.

**Conclusions:**

Our results enlighten the genetic basis of fundamental and putatively virulence-related traits of dermatophytes, advancing future research on these medically important pathogens.

## Background

**Figure 1:**
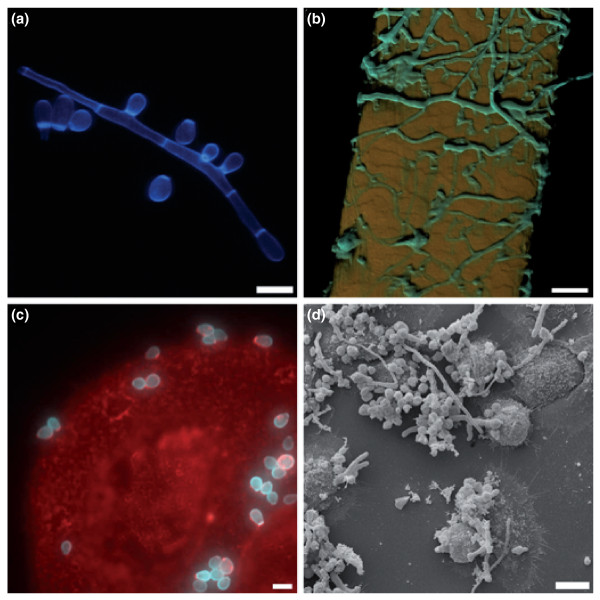
**Hyphae and microconidia of *A. benhamiae *on human hair and human keratinocytes**. **(a) **Fluorescence microscopic picture (laser scanning microscope LSM 5 LIVE, Zeiss, Jena) of hyphae and microconidia stained with fluorescent brightener 28 (Sigma, USA). Scale bar: 5 μm. **(b) **Colonization of human hair. Cyan, fluorescence brightener 28-stained fungal hyphae; orange, hair autofluorescence. Scale bar: 20 μm. **(c) **Attachment of microconidia to human keratinocytes. Cyan, fluorescence brightener 28-stained fungal hyphae, red, wheat-germ agglutinin stained keratinocytes. Scale bar: 5 μm. **(d) **Human keratinocytes with germinating *A. benhamiae *microconidia. Scanning electron microscopy image. Scale bar: 10 μm. See Additional file 1 for supplementary information pertaining to this figure.

Dermatophytes are highly specialized pathogenic fungi and the most common cause of superficial mycoses in humans and animals [1]. During disease, these microorganisms exclusively infect and multiply within keratinized host structures - for example, the epidermal stratum corneum, nails or hair - a characteristic that is putatively related to their common keratinolytic activity [2] (Figure 1; Additional file 1). Consistent with this assumption, during *in vitro *cultivation with keratin as the sole source of carbon and nitrogen, dermatophytes were proven to secrete multiple proteases, some of which have been identified and discussed as potential virulence determinants [2]. Little is known, however, about the general basis of pathogenicity in these fungi, a drawback that might be explained by the fact that these microorganisms have so far not been intensively studied at the molecular level. Dermatophytes are comparatively slow growing under laboratory conditions and genetically less amenable than other clinically relevant fungal pathogens such as *Candida albicans *or *Aspergillus fumigatus *[3]. Recent advances in dermatophyte research allowed the first broad-scale transcriptional and proteomic analyses [4-8], and some selected genes have been functionally characterized [9-11]. However, genome-wide analyses have been hampered by a lack of full genome sequences, thereby precluding the generation of principle hypotheses on dermatophyte pathogenicity in a comparative genomic context.

The two dermatophyte species *Arthroderma benhamiae *and *Trichophyton verrucosum *are both zoophilic, yet the natural reservoir of *T. verrucosum *is almost exclusively cattle, whereas *A. benhamiae *is usually found on rodents, in particular guinea pigs [12,13]. The two species also differ in their ability to grow under laboratory conditions, with *T. verrucosum *being very difficult to cultivate at all [14]. Conversely, *A. benhamiae *is comparatively fast growing and produces abundant microconidia. As a teleomorphic species, the fungus is even able to undergo sexual development, including the formation of sexual fructifications (cleistothecia) [15,16]. These characteristics, together with the recent establishment of a guinea pig infection model and a genetic system for targeted gene deletion (P Staib and colleagues, manuscript submitted) for this species, suggest *A. benhamiae *is a useful model organism to investigate the fundamental biology and pathogenicity of dermatophytes [8]. Despite the above mentioned phenotypic differences, *A. benhamiae *and *T. verrucosum *are phylogenetically very closely related, and both induce highly inflammatory cutaneous infections in humans, such as tinea corporis [15,17]. Therefore, a genome comparison of the two species should reveal common basic pathogenicity-associated traits.

In the present study, we report and compare the genome sequences of *A. benhamiae *and *T. verrucosum *and refer to potential dermatophyte-specific pathogenicity-associated factors, as revealed by comparisons with groups of proteins important for pathogenicity in other species of the Onygenales (*Coccidioides posadasii *and *Coccidioides immitis*) and in the mould *A. fumigatus*. Applying our insights thereof, we used secretome analysis to reveal secreted factors of *A. benhamiae *that mediate extracellular *in vitro *keratin degradation. The interaction between *A. benhamiae *and the human host was monitored by global transcriptome profiling of the fungal cells in contact with human keratinocytes. Investigating the molecular basis of sexual reproduction, we inspected in detail the *A. benhamiae *mating type locus.

## Results and discussion

### Comparative genomics of *A. benhamiae *and *T. verrucosum*

The genomes of *A. benhamiae *and *T. verrucosum *were sequenced by a whole-genome shotgun hybrid approach. The assembly of *A. benhamiae *spans 22.3 Mb [DDBJ/EMBL/GenBank:ABSU00000000] and that of *T. verrucosum *comprises 22.6 Mb [DDBJ/EMBL/GenBank:ACYE00000000] (Table 1; Additional file 2; both genomes are also deposited in the Broad Institute database [18]). Thus, these genomes are smaller than those of phylogenetically related ascomycetes, such as aspergilli (28 Mb and 37.3 Mb in case of *Aspergillus clavatus *and *Aspergillus niger*, respectively), *Coccidioides *species (27 to 29 Mb), and *Histoplasma capsulatum *(30 to 39 Mb).

**Table 1:** Genome data of *A. benhamiae *and *T. verrucosum*

	Length (Mb)	Predicted CDS	Mean CDS length	Genes with introns	Predicted tRNAs
*A. benhamiae*	22.3	7,980	1,482	5,809	80
*T. verrucosum*	22.6	8,024	1,458	5,744	77

CDS, coding sequence.

**Figure 2:**
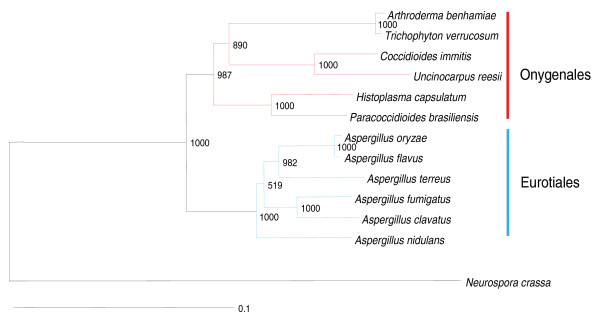
**Partial genome-based phylogenetic tree of *A. benhamiae *and *T. verrucosum *representing the most closely related clades**. The tree was inferred by the neighbor-joining analysis method using the PHYLIP package [59], with the number of bootstrap trials set to 1,000. Numbers at the nodes indicate the bootstrap support. See the details and the entire tree in Additional file 3.

The genomes of *A. benhamiae *and *T. verrucosum *contain 7,980 and 8,024 predicted protein-encoding genes, respectively (Table 1). Introns were found in 5,809 of the *A. benhamiae *and 5,744 of the *T. verrucosum *genes. Both genomes comprise a mosaic of long G + C rich, gene-containing portions separated by A + T rich 'islands' with a GC content below 40%, ranging from a few kilobases to more than 25 kb. As expected from previous reports based on nuclear ribosomal internal transcribed spacer regions 1 and 2 [15,19-21], the comparison of the two genome sequences revealed a strong similarity. Using the software Mummer [22], approximately 21.8 Mb of the genomes (98.0% of the available *A. benhamiae *and 96.7% of the *T. verrucosum *genomic sequences) can be aligned to each other, indicating that the vast majority of genes lie in collinear regions and are shared between the two organisms. The average identity of the alignable portion of the genomes is 94.8%. The alignment of the two genomes points to only five major genomic rearrangements, one inversion and four balanced translocations between chromosomes (Figure S1 in Additional file 2). The presence of only a few rearrangements between the two genomes suggests very recent speciation. These findings are reflected by the phylogenetic tree constructed by use of the available genome sequences (Figure 2; Figure S2 in Additional file 3).

However, we also identified notable dissimilarities between the genomes of *A. benhamiae *and *T. verrucosum*. After having detected the orthologous pairs with best bidirectional hits, we came up with lists of proteins that presumably were unique for either species. Since the best bidirectional hits were identified using protein Blast, we next applied BlastN to correct for possible gene prediction errors. We used a filter threshold for significant hits of 80% identity between sequences over less than 50% of the query length. There were 238 *A. benhamiae *sequences that gave no hits or non-significant hits in *T. verrucosum*, and 219 *T. verrucosum *genes were not found in *A. benhamiae*. Of these, 83 and 78 genes (*A. benhamiae *and *T. verrucosum*, respectively) have assigned names and/or functional domains. A list of the predictions is provided in Additional file 4. Given the overall strong genome sequence similarity, a future functional investigation of these distinctions appears to be of interest, in particular with respect to the tremendous differences between the two species in terms of *in vitro *growth ability and animal host preference (see also the 'Other interesting genes' section).

We analyzed the *A. benhamiae *fast-evolving genes in comparison to *T. verrucosum*. Using the dN/dS ratio as a measure for selective pressure, we obtained a list of positively selected genes (dN/dS >1) (Additional file 5). In total we found 132 positively selected genes with assigned functions, enabling assumptions about their roles in the cell and, hence, the reasons for their accelerated evolution. Of particular interest are the two most abundant groups of these genes, those encoding transcription factors (18 genes) and MFS transporters (5 genes). The latter are known to be usual constituents of secondary metabolite (SM) gene clusters.

Both dermatophyte genomes encode the basic metabolic machinery for glycolysis, tricarboxylic acid cycle, glyoxylate cycle, pentose phosphate shunt, and synthesis of all 20 standard amino acids and the five nucleic acid bases. Moreover, dermatophytes appear to be capable of producing a wide range of SMs, which is reflected by the presence of polyketide synthase (PKS)- and non-ribosomal peptide synthetase (NRPS)-encoding genes (see the 'Genetic basis for secondary metabolism gene clusters' section). The outstanding ability of dermatophytes to specifically infect superficial host structures may be supported by the possession of a broad repertoire of genes encoding hydrolytic enzymes, the expression of many of which was also proven experimentally (see the next paragraph and the 'Identification of secreted fungal proteins during keratin degradation by secretome analysis' section). In addition, the ability of dermatophytes to assimilate lipids, major constituents of the skin, is putatively reflected by the presence of 16 lipase genes in either genome. A putative link between the possession of lipases and fungus-induced skin disease has previously been revealed for basidiomycetes of the genus *Malassezia *[23].

Of particular note is the apparent relative paucity of tRNA genes in both dermatophytes in comparison with other closely related ascomycetes. The genomes of *A. benhamiae *and *T. verrucosum *contain 80 and 77 tRNA genes, respectively, whereas the number of tRNA genes varies between approximately 100 to 130 in *Coccidioides *species and 150 to 370 in aspergilli. However, some strains of *H. capsulatum*, representing a comparatively closely related pathogen, also possess only 83 to 89 tRNA genes, suggesting that the low number of tRNA genes is not specific to dermatophytes.

### Identification of a broad repertoire of protease genes in dermatophyte genomes

Dermatophytes are keratinophilic fungi, sharing the ability to utilize compact hard keratin as a sole source of carbon and nitrogen. In line with this knowledge, the two sequenced genomes reflect a remarkable metabolic capability for protein degradation. They contain 235 predicted protease-encoding genes, 87 of the deduced proteins possessing a secretion signal (Table S3 in Additional file 6). We did not detect any protease in *A. benhamiae *or *T. verrucosum *unique to either species, a finding that may reflect similar life styles and/or host adaptation mechanisms, especially with respect to their common keratinophilic growth. In general, deviations in the number of proteases per genome are rather large in the fungal kingdom, ranging from approximately 90 in *Ustilago maydis *to approximately 350 in *Gibberella zeae *(according to the MEROPS database [24]). Dermatophytes belong to the most protease-rich species.

The protein sequence of each protease is highly conserved across dermatophyte species [25]. Collections of predicted secreted proteases of *A. benhamiae *and *T. verrucosum *as well as *Coccidioides *spp. (Onygenales) were compared to those of *A. fumigatus *as a member of the Eurotiales, for which many secreted proteases have previously been characterized. Most *A. fumigatus *proteases in A1 (pepsins), M28 (leucine aminopeptidases), S9 (dipeptidylpeptidases), S10 (carboxypeptidases) and S53 (tripeptidylpeptidases) families have an orthologue in dermatophytes and *Coccidioides *spp. (Table S4 in Additional file 7). The major striking differences found between the secreted protease batteries of *A. fumigatus *and Onygenales are the following: subtilisin (S8), deuterolysin (M35), and fungalysin (M36), which belong to endoprotease gene families, have expanded in Onygenales (Table S4 in Additional file 7); the same is true for exopeptidases of the M14 family (metallocarboxypeptidases) and the M28 family (aminopeptidases) - a major carboxypeptidase (McpA) homologous to the human pancreatic carboxypeptidase A was previously characterized in dermatophytes [26], and of particular note, *Aspergillus *spp. have no McpA orthologue; and genes encoding acidic glutamic proteases (G1 family) were not detected in either dermatophytes or *Coccidioides *spp.

Major differences between dermatophytes and *Coccidioides *spp. proteases were found in M35, M36 and S8 proteases families (see the phylogenetic trees in Additional file 8). Proteases of these three families of dermatophytes and *Coccidioides *spp. form distinct clades in phylogenetic trees (Additional file 8). Members of the S8 and M36 families have undergone additional amplifications in the dermatophyte lineage, and expansion of the M35 family appears to be different in *Coccidioides *spp. and dermatophytes. In the latter, a clade was apparently lost. In addition, three genes encoding proteases of the S41 family were found in the dermatophyte genomes while only one gene encoding a protease of this family was identified in *Coccidioides *spp.

Recent comparative genomic analyses of *Coccidioides *species with other members of the Onygenales showed gene family sizes are associated with a host/substrate shift from plants to animals in these microorganisms [27]. Experimentally, the expression of genes encoding fungalysins and subtilisins was recently monitored in *A. benhamiae *by cDNA microarray analysis during growth on keratin, and also during cutaneous infection of guinea pigs [8]. Interestingly, the prominent keratin induced *A. benhamiae *subtilisin-encoding genes, such as *SUB3 *and *SUB4*, were not observed in this former analysis to be strongly activated *in vivo*, in contrast to others that conversely were not found to be induced during *in vitro *growth on keratin. A role for Sub3 was recently observed in adhesion of the dermatophyte *Microsporum canis *to feline epidermis, but not for the invasion thereof [28]. These findings suggest additional functions of secreted proteases during host adaptation other than keratin degradation. Since the formerly used cDNA microarray does not comprise the full genome of *A. benhamiae*, the future identification of *in vivo *specific dermatophyte proteases on the basis of the presented genome appears to be of major interest.

### **Identification of secreted fungal proteins during keratin degradation by secretome analysis**

**Figure 3:**
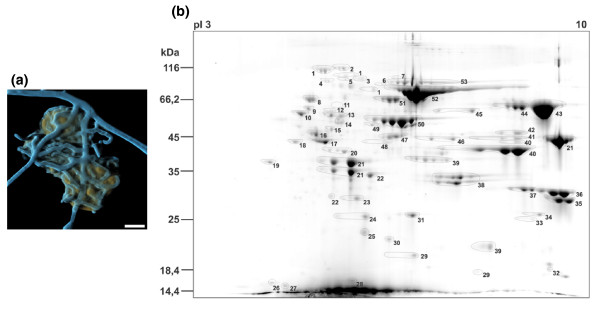
**Secretome of *A. benhamiae *grown on keratin**. **(a) ***A. benhamiae *grown on keratin particles. Cyan, fluorescence brightener 28-stained fungal hyphae; orange, keratin particle autofluorescence. Scale bar: 10 μm. **(b) **Two-dimensional gel of secreted *A. benhamiae *proteins obtained from culture supernatant after 48 h cultivation in a shaking flask with 0.9 g/l glucose and 10 g/l keratin. The apparent molecular mass of proteins and the pI range of the first dimension are indicated. Proteins were identified by mass spectrometry (matrix-assisted laser desorption/ionization-time of flight/time of flight (MALDI-TOF/TOF)). Identified proteins are given in Table S5 in Additional file 6. See also Additional file 1 for more details.

A potential role of secreted proteases, in particular serine proteases, in pathogenesis has been widely reported in many prokaryotes and fungi [2,29-31], including functions as allergens [32]. In order to apply insights from the present genome sequences to determine putative virulence gene function, we set out to reveal the basic panel of factors that are secreted during growth of *A. benhamiae *on keratin. To achieve this, secretome analysis was performed, an approach that, to our knowledge, has not been applied to *A. benhamiae *before. Experimental analysis (after 2 days of growth) led to the identification of 203 single electrophoretic species (Figure 3b). From these entities, 53 different proteins were detected (Table S5 in Additional file 6). By far the largest group of identified proteins is formed by putative proteases (approximately 75% relative spot volume). In addition, we found other, different hydrolases and proteins involved in carbohydrate metabolism (Table S5 in Additional file 6). Three of the subtilisin-like serine proteases (Sub3, Sub4, and Sub7), three fungalysine-type metalloproteases (Mep1, Mep3, and Mep4), the leucine aminopeptidases Lap1 and Lap2, as well as the dipeptidyl-peptidases DppIV and DppV were detected in the secretome, consistent with gene expression analysis in *A. benhamiae *during keratin degradation [8]. Supporting our results, the pattern of proteins secreted by the two related dermatophyte species *Trichophyton rubrum *and *T. violaceum *during growth on soy protein was previously described in [4]. In that study, a gel-based approach led to the identification of 19 proteins secreted by at least one of these species. Remarkably, 15 of the corresponding homologs were also found to be secreted in the present study by *A. benhamiae *on keratin medium, including major keratinases of the subtilisin family of secreted proteases (also see Table S6 in Additional file 6). Individual differences between the present and formerly observed secretion patterns might be due to the different dermatophyte species analyzed and/or to the different protein substrates and cultivation parameters used. In conclusion, the set of dermatophyte secreted proteases in a protein medium is similar to that of *A. fumigatus*, which includes endoproteases such as the major subtilisin Alp1 and the fungalysin Mep and exoproteases such as Lap1, Lap2, DppIV and DppV.

Endo- and exoproteases secreted by microorganisms cooperate very efficiently in protein digestion to produce oligopeptides and free amino acids that can be incoporated via transporters. During the process of protein digestion the main function of endoproteases is to produce a large number of free end peptides on which exoproteases may act. At neutral and alkaline pH, synergistic action of Lap and DppIV was shown in *Aspergillus *spp. [13,24]. Laps degrade peptides from the amino terminus until reaching an X-Pro sequence, which acts as a stop. In a complementary manner, the X-Pro sequences can be removed by DppIV, thus allowing Laps access to the next residue. Dermatophyte and *Aspergillus *spp. Lap1, Lap2, DppIV and DppV have shown comparable substrate specificity [33]. Therefore, our proteomics approach allows us to hypothesize common basic mechanisms in dermatophytes during extracellular protein digestion. However, the presence of large protease gene families in dermatophytes reflects selection during evolution and the ability of these fungi to adapt to different environmental conditions during infection and saprophytic growth.

### Differential gene expression in *A. benhamiae *during infection of keratinocytes

Growth of *A. benhamiae *on keratin might mimic selected *in vivo *growth substrates, yet may not reflect the entire process of infection. In order to gain more insights into basic host adaptation mechanisms, we studied the global transcriptional response of *A. benhamiae *during infection of human keratinocytes. After 12 h of co-cultivation, germinating *A. benhamiae *microconidia were observed to be localized and concentrated on the host cells, suggesting that the fungus actively adheres to the keratinocytes (Figure 1c,d). To perform 454 RNA sequencing, the fungal cells were harvested after incubation for 96 h with and without keratinocytes. About 50 *A. benhamiae *genes showed differential expression with a fold change >5 (*P*-value < 0.05; Table S7a in Additional file 6); 45 genes encoding putatively secreted proteins (Table S5 in Additional file 6) and 13 genes coding for proteins involved in the biosynthesis of SMs are expressed either only with or without keratinocytes, or under both conditions. Of the 235 predicted protease-encoding genes, 158 are expressed under both conditions. Sixteen potentially secreted proteins, including three proteases, are differentially expressed (Table S7b in Additional file 6). In particular, the expression profile of the genes encoding carboxypeptidase S1 and dipeptidyl-peptidase DppV implies their potential involvement in the infection process. The transcript levels of two NRPS genes were reduced during co-cultivation with keratinocytes, a finding that is noticeable but cannot be explained at this stage.

To confirm the RNAseq results, we selected several genes that were predicted to be differentially expressed and tested them by Northern blotting. We used two housekeeping genes, actin (ARB_04092) and glyceraldehyde 3-phosphate dehydrogenase (GAPDH, ARB_00831), as controls as they are not expected to be differentially regulated between the control and co-incubation conditions. All tested genes were regulated as expected from the RNAseq data (Figure S4 in Additional file 9). The expression level alterations of metabolic enzymes (ARB_07891, ARB_04156, ARB_01650 and ARB_04856) and membrane transporters (ARB_01027) reflect the adaptation of the fungus to the different nutrition provided by keratinocytes and their remnants, whereas the strong up-regulation of the hydrophobin ARB_06975 indicates altered binding properties and adhesivity during growth on epithelial cells and during infection. In conclusion, this independent experimental method shows that the accuracy of the RNAseq data was exemplary.

### Genetic basis for secondary metabolism gene clusters

**Figure 4:**
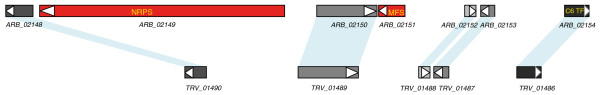
***A. benhamiae *NRPS ARB_02149 gene cluster and the corresponding region in the *T. verrucosum *genome**.

The *A. benhamiae *and *T. verrucosum *genomes encode a relatively high number (26 and 25, respectively) of SM biosynthesis gene clusters (Table 2), a finding that contrasts with observations made in other fungi and bacteria highly adapted to humans. For comparison, *Candida albicans *or *Staphylococcus aureus *hardly produce SMs and *Histoplasma *species have no more than seven SM gene clusters per genome; more closely related to dermatophytes is *Coccidioides immitis*, which has 16 SM gene clusters, the main difference being in the number of NRPSs (5 versus 15 in *A. benhamiae*). Nine PKS, 15 NRPS and 3 PKS/NRPS hybrid genes were identified in the *A. benhamiae *genome, all of which except for one NRPS gene (ARB_02149) are conserved in both species (Table 2). Addressing the question of whether the absence of the latter gene in *T. verrucosum *is associated with phenotypic and/or host-specific differences between the two species will be of future interest. To see whether only the NRPS or the entire associated gene cluster is absent from *T. verrucosum*, we examined the conservation of the other constituents of the ARB_02149 gene cluster and observed that the 'missing' NRPS belongs to an otherwise very well conserved and collinear region that spans more than 75 kb (the whole *T. verrucosum *supercontig 79). However, one other gene besides ARB_02149 is missing in *T. verrucosum*, the MFS transporter ARB_02151 (Figure 4). Interestingly, the 'missing' genes are separated by a perfectly conserved ABC multidrug transporter (ARB_02150 = TRV_01489). The *Arthroderma *ARB_02149 gene cluster has several traits typical of functional SM gene clusters, such as the presence of genes for the MFS transporter, feruloyl esterase and C6 transcription factor. This makes us suppose that the NRPS was lost in *Trichophyton *rather than acquired by *Arthroderma*. However, it remains unclear if the MFS transporter was deleted simultaneously, and why the deletion did not capture the 'middle' ARB_02150 gene.

**Table 2:** Putative PKS and NRPS genes of *A. benhamiae*, *T. verrucosum*, and *C. immitis*

Type	LocusLink *Arthroderma benhamiae*	LocusLink *Trichophyton verrucosum*	LocusLink *Coccidioides immitis*	Domain architecture
PKSs				
Non-reducing	ARB_00538	TRV_00386	-	KS-AT-ACP
	ARB_03291	TRV_02519	CIMG_13102	KS-AT-ACP-ME^a^
	-	-	CIMG_05571	KS-AT-ACP
	-	-	CIMG_04689	KS-AT-ACP-ME
	-	-	CIMG_03162	KS-AT-ACP
	ARB_07994	TRV_04611	CIMG_08569	KS-AT-ACP-ACP-TE
	-	-	CIMG_08564	AT-KS-ACP-TE
Reducing	ARB_01525	TRV_04236	CIMG_13632	KS-AT-ME-ER-KR-ACP
	ARB_05854	TRV_06867	-	KS-AT-KR-ACP^b^
	ARB_06393	TRV_01071	-	KS-AT-ME-ER-KR-ACP
	ARB_05333	TRV_06912	CIMG_02398	KS-AT-DH-MT-ER-KR-ACP
	ARB_07933	TRV_04104	-	KS-AT-ME-ER-KR-ACP
	ARB_07966	TRV_04285	-	KS-AT-ME-KR-ACP
	-	-	CIMG_05569	KS-AT-DH-ER-KR-ACP
	-	-	CIMG_03014	KS-AT-DH-ER-KR-ACP
	ARB_00195	TRV_05651	CIMG_07298	A-T-C-T-C
	-	-	CIMG_01429	A-T-C-T
	ARB_01698	TRV_01735	CIMG_09750	C-A-T-C-A-T-C-A-T-C-A-T-C-A-T-C-T-C-T
	ARB_02149	-	-	C-A-T-C-A-T-C-A-T-C-A-T-C^c^
	ARB_02226	TRV_00553	-	A-T-C-A-T-C-A-T-C
	ARB_02570	TRV_5508	-	A-T-C
	ARB_02750	TRV_06186	-	A-T-C-A-T-C-A-T-C-A-T-C-A-T-C-T
	ARB_03095	TRV_06056	-	T-C-A-T-C/T-C-A
NRPSs	ARB_03768	TRV_07570	-	A-C-A-T-C-A-T
	ARB_04984	TRV_06313	CIMG_01861	A-T-C-A-T-C
	ARB_05131	TRV_07837	-	A-T-C-A-T-C-A-T
	ARB_05579	TRV_06828	-	T-C-A-T-C-A-T
	ARB_06786	TRV_05681	-	A-T-C
	ARB_07686	TRV_05452	CIMG_00941	A-T-C-A-T-C-T-C-A-T-C-T-C-T-C
	ARB_07850	TRV_01776	-	A-T-C/A-T-C-A-T
	ARB_07862	TRV_04720	-	A-T-C-A-T-C-T
	ARB_07534	TRV_00508	-	KS-AT-DH-ER-KR-ACP-C-A-T
PKS/NRPS hybrids	ARB_02973	TRV_03721	CIMG_06629	KS-AT-ME-KR-ACP-C-A-T
	ARB_07844	TRV_05146	-	A-T-KS-AT-KR-ACP-TE

^a^Potential citrinin-like product; similar to pksCT BAD44749.1. ^b^Product 6-methyl-salicylic acid; similar to 6-MSA synthase CAA39295.1. ^c^Unique for *A. benhamiae*. A, adenylation domain; ACP(PP), acyl carrier protein, or phosphopantetheine domain; AT, acetyltransferase domain; C, condensation domain; DH, dehydratase domain; E, epimerization domain; ER, enoyl reductase domain; KR, ketoacyl reductase domain; KS, beta-ketoacyl synthase domain; ME, methyltransferase domain; T, thiolation domain; TE, thioesterase domain.

All nine PKS genes detected in *A. benhamiae *have unequivocal counterparts in the *T. verrucosum *genome (Table 2). An interesting feature of the dermatophyte PKS set is the unusual proportions of reducing and non-reducing PKSs. Whereas in all other closely related ascomycetes (such as aspergilli) most of the PKSs are non-reducing, in dermatophytes most are reducing PKSs. A comparison with the closest sequenced relative, *C. immitis *(Table 2; see more details below), also revealed substantial differences in the composition of the PKS set: the ratio of reducing to non-reducing in dermatophytes is 2:1, whereas in *C. immitis *it is 2:3. This observation suggests dermatophytes have an uncommon SM profile, which deserves future investigation. Particular attention should be paid to the fact that these fungi are characterized by intense pigmentation, a phenotype that may be related to their pathogenicity. For the related species *T. rubrum*, the polyketide-derived mycotoxin xanthomegnin has been suggested to be responsible for the characteristic red colony reverse pigment. Most interestingly, xanthomegnin production has even been detected in epidermal material infected by *T. rubrum*, in contrast to non-infected controls [34]. A putative link between SM production and host adaptation of *A. benhamiae *might also be reflected by our observation that several genes associated with the synthesis of such molecules were found to be differentially regulated during infection of human keratinocytes (see the 'Differential gene expression in *A. benhamiae *during infection of keratinocytes' section).

To get an impression of possible expansions of families and evolutionary relationships, we compared the sets of SM producers in dermatophytes with that of *C. immitis *(Table 2; Figure S5.1 and S5.2 in Additional file 10). As mentioned above, the total number of SM gene clusters is higher in dermatophytes, mainly due to the more abundant NRPSs. However, we observe differences also in the PKS set as well as in the number of PKS/NRPS hybrids: *C. immitis *possesses only one hybrid, whereas each dermatophyte has three. The higher number of non-reducing PKSs in *C. immitis *is mainly due to the expansion of one clade; most likely we are seeing the results of duplication of some ancestor genes with a domain architecture of a beta-ketoacyl synthase domain, an acetyltransferase domain, an acyl carrier protein domain, and a methyltransferase domain (KS-AT-ACP-ME). Four of six *C. immitis *non-reducing PKSs belong to this clade. Of the other two, one has a clear ortholog in dermatophytes, and the other has an unusual structure (AT-KS-ACP-thioesterase domain (TE)) without an orthologous dermatophyte gene. In comparison to *C. immitis*, dermatophytes possess two additional non-reducing clades, which means that, in spite of the lower number of non-reducing PKSs, they have more various potential capacities. The reducing *C. immitis *PKSs also cannot boast great variety: two of four *C. immitis *genes are most likely the result of a duplication (they form a separate clade and do not have dermatophyte orthologs), one PKS has orthologs in dermatophytes, and one is only a probable homolog (see below). On the other hand, in dermatophytes we see an expansion of the group with a fumonisin synthase-like structure (KS-AT-ME-enoyl reductase domain (ER)-ketoacyl reductase domain (KR)-ACP): three orthologous pairs formed by out-paralogs in each species have only one close homolog in *C. immitis*. Since the *C. immitis *gene lacks one of the domains (methyltransferase), we cannot consider it as a fumonisin-like ortholog. Besides the 6-methyl-salicylic acid synthase, completely lacking in *C. immitis*, another not completely reducing PKS (KS-AT-ME-KR-ACP), as well as two PKS/NRPS hybrids, do not have homologs in *C. immitis*. Taken together, these data agree with our hypothesis that highly adapted parasites such as *Coccidioides *do not require a large arsenal of SMs.

### Sexuality in dermatophytes

**Figure 5:**
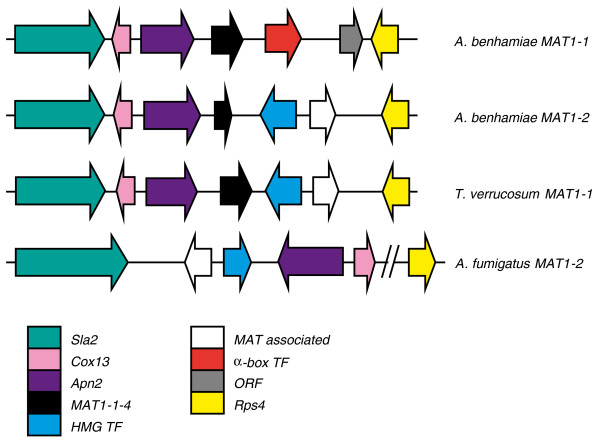
**Mating type gene organization of *A. benhamiae *and *T. verrucosum***. Genes constituting the MAT locus: *Sla2*, putative cytoskeleton assembly control protein (ARB_07317, TRV_02048, AFUA_3G06140); *Cox13*, cytochrome C oxidase subunit VIa (ARB_08059, TRV_08208, AFUA_3G06190); *Apn2*, DNA lyase (ARB_07318, TRV_02049, AFUA_3G06180); a gene similar to *MAT1-1-4 *(ARB_07319, TRV_02050); HMG TF, HMG-box transcription factor (*MAT1-2-1*; ARB_7320, TRV_02051, AFUA_3G06170); *MAT *associated protein of unknown function (ARB_07321, TRV_02052, AFUA_3G06160); α-box transcription factor (*MAT1-1-1*, GB GQ996965); *ORF*, glycine rich protein of unknown function; *Rps4*, protein S4 of the 40S ribosomal subunit (ARB_7322, TRV_02053, AFUA_3G06840).

Sexual reproduction is known for *A. benhamiae *but not for *T. verrucosum *[35,36]. The *A. benhamiae *and *T. verrucosum *genomes revealed the whole sets of genes for mating and meiosis in both species, suggesting that the lack of a known sexual cycle in *T. verrucosum *is not due to major deletions of genes essential for sexual reproduction and meiosis (Table S8 in Additional file 6). Both sequenced strains showed a single mating type encoding an HMG box transcription factor. To identify the complementary mating type, we sequenced the corresponding region of an *A. benhamiae *mating partner strain (strain CBS 809.72; Figure 5). The newly identified region encodes an alpha-box type transcription factor, indicating that *A. benhamiae *exhibits two mating types, as described for other closely related fungal pathogens such as *H. capsulatum *and *C. immitis *[37]. *A. benhamiae *mating type + strains as well as mating type - strains are often routinely isolated [36]. There is no apparent disequilibrium between mating type + and mating type - strain frequencies.

We did not identify a striking defect in the *T. verrucosum *mating type locus, which appears to be intact. Several strains of *T. verrucosum *were found to be of the same mating type as the sequenced strains, suggesting a strong disequilibrium towards mating type +.

In *Aspergillus *(Eurotiales), *Coccidioides *and *Histoplasma *(Onygenales) the mating type (MAT) loci are flanked by *APN2 *and the *SLA2 *genes encoding a DNA lyase and a cytoskeleton protein, respectively [37]. The MAT idiomorphs and flanking regions described here for *A. benhamiae *and *T. verrucosum *are essentially identical to those of other closely related dermatophytes [38].

### Other interesting genes

Of particular interest are the genes of *A. benhamiae *that have no obvious counterpart in *T. verrucosum *(Additional file 4) and whose predicted functions suggest their potential involvement in basic biological phenotypes and/or pathogenicity. Two such genes, ARB_04713 and ARB_02149, encoding a phosphopantetheine-binding domain and an NRPS, respectively, were found in the transcriptome analysis, although not expressed differentially. The expression pattern of the *A. benhamiae*-specific NRPS ARB_02149 further suggests that its as yet unidentified product is produced during infection by the fungal cells.

Another gene of particular interest encodes hydrophobin. In *A. fumigatus*, surface hydrophobin was shown to prevent immune recognition [39]. The *A. benhamiae *hydrophobin gene (ARB_06975) shows 99% similarity with the respective *T. verrucosum *gene (TRV_00350) and displays moderate overexpression (1.6×) under co-cultivation conditions (Table S7b in Additional file 6). The analysis of a potential role of dermatophyte hydrophobins in immune response functions and/or adhesion to host surfaces will be part of future research.

## Conclusions

Numerous examples in microbial pathogenicity research still need to be explained at the genomic level, thus requiring genome sequences to be made available. Here, we present the first genomes of dermatophyte species, filamentous fungi that cause most superficial infections in humans and animals. The presence of putative pathogenicity-related factors, such as numerous secreted proteases, was revealed at the genome level and also experimentally confirmed during keratin degradation by *A. benhamiae*. Although keratin utilization is traditionally supposed to be of major relevance for the pathogenicity of these microorganisms, the entire process of host adaptation during infection seems to be more complex. Transcriptome analysis showed that only some of the typically keratin-induced proteases were found to be strongly expressed during fungus-keratinocyte interaction. Instead, genome and transcriptome analyses draw attention to so far hardly noticed dermatophyte factors - for example, putative SMs - the role of which should be addressed in the future. Our research on dermatophytes was strongly facilitated by the selection of *A. benhamiae *as a model species, which provides practical advantages such as comparatively fast growth and the production of abundant microconidia. Moreover, future basic studies on the regulation of mating, dermatophyte evolution and host preference will profit from the ability of *A. benhamiae *to undergo sexual reproduction. In conclusion, by presenting dermatophyte genomes and global insights into major processes of host adaptation, we intend to advance molecular studies on these medically important microorganisms.

## Materials and methods

### *A. benhamiae *and *T. verrucosum *strains and growth conditions

A clinical isolate of *A. benhamiae *strain 2354 was used (isolate LAU2354) [15]. *T. verrucosum *strain 44 [17] was kindly provided by Yvonne Gräser (Charité, Berlin, Germany). Strains were cultivated at 28°C on Sabouraud 2% (w/v) glucose agar (SG, Merck, Darmstadt, Germany) for 12 days; liquid cultures were shaken at 180 rpm at 30°C for 5 to 7 days. Hyphae and conidia were separated by filtration using a 40 μm cell strainer (BD Bioscience, Heidelberg, Germany). Conidia were counted with a cell counter (Beckman, Coulter, Krefeld, Germany) or manually using a Thoma chamber. For crossing experiments of *A. benhamiae *LAU2354 with the opposite mating type CBS 809.72, MAT medium [40] (1/10 SG, 0.1% (w/v) MgSO_4 _and 0.1% (w/v) KH_2_PO_4_) was used.

### DNA and RNA preparation for DNA sequencing and cDNA library

For DNA preparation, mycelia were separated from the medium by filtration through Miracloth (Calbiochem, Darmstadt, Germany) and ground in a mortar under liquid nitrogen. After evaporation, the powder was suspended in a solution containing 150 mM EDTA, 50 mM Tris-HCl, pH 8.0, 1% (w/v) SDS, 20 mM NaCl and 100 μg/ml proteinase K (Merck). After incubation for 1 h at 55°C, the solution was gently mixed with 1/4 volume of 4 M NaCl and kept on ice for 30 minutes. After centrifugation for 10 minutes at 6,000 rpm and 4°C, polyethylene glycol 6000 (Serva, Heidelberg, Germany) was added to the supernatant to a final concentration of 10% (w/v). The DNA was precipitated for 1 h on ice and centrifuged for 10 minutes at 10,000 rpm at 4°C. The pellet was dissolved in a solution containing 25 mM Tris-HCl, pH 8.0, 5 mM EDTA, 10 mM NaCl and 1% (v/v) Triton X100. For density centrifugation, 1g CsCl and 12 μl bisbenzimide (10 mg/ml) for each milliliter of solution were added [41,42]. Ultracentrifugation was performed in a vertical rotor at 44,000 rpm for 24 h at 25°C. DNA was separated into two bands of different density according to the AT-content of the DNA. The upper band contained a DNA fraction highly enriched for mitochondrial DNA. For *T. verrucosum*, two rounds of density gradient centrifugation were necessary. In the first round, ethidium bromide was used instead of bisbenzimide. For RNA preparation, SG medium was inoculated with conidia to a final concentration of 3 × 10^4 ^conidia/ml and shaken at 180 rpm for three days at 30°C. Total RNA was isolated using a commercial kit as described by the manufacturer (Qiagen, Hilden, Germany). After RNA extraction, a cDNA library was constructed from this material according to the manufacturer's protocols (MINT cDNA synthesis kit, Evrogen, Moscow, Russia).

### Plasmid/fosmid libraries and sequencing

Nuclear DNA of *A. benhamiae* and *T. verrucosum* was sheared, size fractionated (3 to 4 kb), end-repaired, and cloned into the *Sma*I site of pUC18. For both fungal species, two fosmid libraries each were prepared in pCC1FOS (Epicentre Biotechnologies, Madison, WI, USA) as described by the manufacturer, one for the high-GC chromosomal DNA fraction and one for the AT-rich mitochondrial DNA fraction. For *T. verrucosum*, 40,000 fosmids from GC-rich and 80,000 fosmids from AT-rich DNA were obtained. For *A. benhamiae*, the corresponding yields were around 50,000 (GC-rich) and 20,000 (AT-rich), respectively. End sequences of plasmid and fosmid clones were obtained using dye terminator chemistry and a 3730×l sequencer (Applied Biosystems, Foster City, CA, USA). Moreover, a fosmid library was generated with a GC-rich DNA fraction of the *A. benhamiae *strain CBS 809.72 encoding the opposite mating type locus. We tested 1,000 fosmids by colony filter hybridization and in PCR experiments. Fosmids were identified by hybridization experiments with a digoxygenin-labeled part of the *apn2 *gene (ARB_07318) and in PCR experiments using *apn2 *amplifying primers (5'-CTTCTAGTGACTCGCCACAGG-3' forward and 5'-GAGTTGGAGGTTGAGATGCTGAC-3' reverse). Three clones were positively identified by both methods. To test whether the fosmids contained the full length MAT region, the clones were tested in PCR experiments amplifying parts of other flanking genes, such as the *sla2 *gene (ARB_07317) and the *rps4 *gene (ARB_07322). For *sla2*, PCR primer pair 5'-CTTGTTCAGGAGAGCTATGG-3' and 5'-CAGCTTCTCGAGCTCCTCCC-3' was used; for *rps4*, PCR primer pair 5'-CAGCGCCTGGTCAAGGTCGACG-3' and 5'-GGTCACGCTCCTCAGCAATGG-3' was used. DNA of a positive fosmid was shotgun sequenced using dye terminator chemistry (ABI).

In addition, genomic 454 libraries were generated according to the manufacturer's protocol and sequenced using a GS FLX (Roche, Mannheim, Germany). The nucleotide sequences were assembled species-specific using the newbler software. Clone gaps were filled using a primer walking strategy with custom primers. Isolation, quantification and quality control of total RNA was performed as described [43]. A cDNA-library was constructed according to the manufacturer's protocols (Evrogen) and 1,411 ABI dye terminator sequences were obtained mainly from the 5' end. The sequences were matched to the assembled genomic sequences to determine exon/intron structures and to obtain an intron signature for the species.

### Next generation sequencing and assembly

The same DNA as for the preparation of the plasmid/fosmid libraries was used for the preparation of genomic libraries for the 454/FLX system (Roche) according to the manufacturer's protocols. Three runs each were performed on the 454/FLX sequencing machine. All 454/FLX sequence data were assembled species-specific using the newbler software. The Sanger based sequencing reads were assembled onto this 'backbone'. Clone gaps were filled using a primer walking strategy with custom primers.

Both genomes are deposited in NCBI with accession codes [DDBJ/EMBL/GenBank:ABSU00000000] for *A. benhamiae *and [DDBJ/EMBL/GenBank:ACYE00000000] for *T. verrucosum*.

### Gene prediction

Gene models of both fungi were generated by using *in silico* predictions and sequence data from an EST library constructed from cultured *A. benhamiae *cells. We matched 1,411 ABI dye terminator sequences obtained from the cDNA library sequencing to the assembled genomic sequences to determine exon/intron structures and to obtain an intron signature for the species. The alignments of the cDNA sequences to the genomic backbone yielded evidence for 861 introns and at least 653 protein-coding open reading frames (coding sequences), which were validated by BLAST. These data were used to train the gene prediction program geneid [44]. To validate the accuracy of the gene prediction, 47 gene structures in one genomic region were annotated manually and compared to the automated predictions, indicating a specificity of 82% at a sensitivity of 97%. For the annotation and comparative analyses of the genomes a web based genome browser was set up using the GenColors database/software system [45].

### Best bidirectional hits and BlastN analysis

Blast analysis of all coding sequences of one genome against the other yielded best bidirectional hits. We used a filter threshold for significant hits of 30% identity between amino acid sequences over at least 50% of the protein.

A BlastN analysis of the genomic sequences was performed for all protein coding genes of *T. verrucosum *against all *A. benhamiae *contigs. A filter threshold for significant hits was 80% identity between sequences over at least 60% of the query length; 239 *T. verrucosum *sequences gave no hits or non-significant hits.

### Transcriptome analysis

The human keratinocyte line HaCaT was obtained from Prof. Fusenig (Deutsches Krebsforschungszentrum, Heidelberg, Germany). The cells were cultivated in DMEM supplemented with 10% (v/v) fetal calf serum, gentamycin (28 μg/ml) and 1% (w/v) ultraglutamine at 37°C in a humidified atmosphere and 5% (v/v) CO_2 _for 2 days. Medium and supplements were purchased from Lonza (Basel, Belgium). Human keratinocytes were infected by *A. benhamiae *conidia with a multiplicity of infection (MOI) of 6. Infected human cells were cultivated in fetal calf serum-free DMEM supplemented with both gentamycin and ultraglutamine for 96 h at 28°C. As a control, *A. benhamiae *conidia were grown in the absence of keratinocytes under the same conditions. After infection, the human keratinocytes were lysed by addition of 0.03% (v/v) Triton X for 2 minutes and *A. benhamiae *was harvested. Fungal cells were collected by centrifugation for 3 minutes at 3,500 g. *A. benhamiae *cells were washed twice in Dulbecco's phosphate buffered saline (Lonza) and stored in aliquots at -80°C. For RNA sequencing, total RNA was isolated using RiboPure™-Yeast Kit (Ambion Europe, Huntingdon, UK) according to the manufacturer's instructions from keratinocytes co-incubated with conidia and conidia only grown in cell culture medium for 96 h.

RNA was reverse transcribed using a SMART technique (Evrogen). The single-stranded DNA was then amplified using SMART primers for 20 cycles to produce double-stranded DNA in sufficient quantity for GS-FLX sequencing (Roche). We generated *A. benhamiae *transcriptome data by sequencing parts of individual cDNAs after fragmentation by nebulization using 454/FLX sequencing technology. For postprocessing of these sequences, SMART adapters were identified and clipped using a combination of perl scripts plus cross_match. After further cleaning with seqclean (removal of polyA tails and low complexity regions), 682,580 ESTs (98.8 Mb) remained for mapping.

Mapping of the ESTs to the repeat-masked *A. benhamiae *genome as a backbone was done in two major steps. First, we used Blat [46] to assign each EST to its most probable position in the genome allowing a maximum intron length of 10 kb. A valid hit required a minimum length of 30 bp and a minimum identity of 90% to the backbone sequence. In the second step, each EST was realigned to its most probable position utilizing a slightly modified version of Exalin [47] that implements the Smith-Waterman algorithm and information theory for better alignments and intron prediction. Using this approach, we were able to align 571,963 ESTs to the genome. Finally, EST positions were translated to positions of known gene models if possible. In this way, we determined for each gene a set of ESTs and thereby its raw expression level. The data were normalized to the total number of mapping ESTs. Table S9 in Additional file 6 shows the total numbers of generated reads, the reads mapped to a genome, and the reads in gene models for each technical replicate of infection and control samples.

The raw counts for the transcripts were analyzed using the R Statistical Computing Environment and the Bioconductor packages DESeq [48] and edgeR [49]. Both packages provide statistical routines for determining differential expression in digital gene expression data using a model based on the negative binomial distribution. The resulting *P*-values were adjusted using the Benjamini and Hochberg's approach for controlling the false discovery rate [50]. Genes with an adjusted *P*-value <0.05 found by both packages were assigned as differentially expressed.

The RNAseq data are submitted to the Sequence read archive of NCBI and are available with the accession numbers [NCBI:SRR070551] and [NCBI:SRR070552] (sample runs) and [NCBI:SRR070553] and [NCBI:SRR070554] (control runs).

### Northern blotting

Total RNA from mycelial samples was isolated using RiboPure™-Yeast Kit (Ambion) according to the manufacturer's instructions. Total RNA was denatured (15 minutes, 60°C; 5% (v/v) formaldehyde, 50% (v/v) formamide, 40 mM MOPS, pH 7) and separated by agarose gel electrophoresis (1.2% agarose, 40 mM MOPS, 10 mM sodium acetate, 2 mM EDTA, 2% (v/v) formaldehyde, pH 7). Blotting, hybridization and chemoluminescent signal detection were performed according to the manufacturer's instructions (DIG Application Manual for Filter Hybridization, Roche). Gel load and blot signal strength were quantified and normalized using Bio-Rad (Munich, Germany) Quantity One (v4.6.7) software.

### Secretome analysis

For cultivation of *A. benhamiae*, medium was prepared as follows: 10 g/l keratin (MP Biomedicals Europe, Illkirch, France) was autoclaved in water and subsequently 20 mM potassium phosphate pH 5.5, 0.4 mM MgSO_4_, 77 mM NaCl, 5 mM glucose and 0.5% (v/v) SL-8 trace elements [51] were added. Microconidia obtained from *A. benhamiae *cultivated for 7 days on MAT agar at 30°C were used to inoculate shaking flasks at a final spore concentration of 10^6 ^per milliliter. After cultivation for 2 days at 200 rpm and 30°C, cultures were filtered through Miracloth (Calbiochem, Darmstadt, Germany) and the supernatant was centrifuged at 4,000 g for 20 minutes at 4°C. Secreted proteins were precipitated with 10% (w/v) trichloroacetic acid/6.5 mM DTT overnight at 4°C. The precipitate was pelleted at 4,000 g for 20 minutes at 4°C and resuspended twice in ice-cold acetone/water (9:1)/6.5 mM DTT followed by subsequent centrifugation steps. The air-dried pellet was dissolved in lysis buffer 3, as described [52]. Immobiline DryStrips of 11 cm covering a pH range from 3 to 10 (GE Healthcare Life Sciences) were rehydrated overnight according to the manufacturer's instructions. Isoelectric focusing was carried out in an Ettan IPGphor II using a 0 to 1 kV gradient for 11 h, 1 to 8 kV for 3 h and finally 8 kV for 24 kVh. Afterwards, strips were incubated for 15 minutes in equilibration buffer (6 M urea, 2% (w/v) SDS, 75 mM Tris^.^Cl pH 8.8, 30% (v/v) glycerol) with 65 mM DTT, followed by an alkylation step of the proteins with 135 mM iodoacetamide in equilibration buffer under the same conditions. Separation of proteins by the second dimension was carried out using pre-cast Criterion gels (12.5% (w/v), Tris-HCl; Bio-Rad) according to the manufacturer's instructions. Proteins were visualized by Colloidal Coomassie Brilliant Blue G-250 staining [53].

### Protein identification

Protein spots were excised from the gels and digested with sequencing-grade Trypsin (Promega, Mannheim, Germany) as described elsewhere [54]. Eluted peptides were mixed with an equal amount of a saturated alpha-cyano-hydroxycinnamic acid (Bruker Daltonics, Bremen, Germany) solution in aqueous 30% (v/v) acetonitrile and spotted on an MTP anchor-chip 800/384 (Bruker Daltonics). Mass spectrometry spectra were acquired with an Ultraflex I TOF/TOF (Bruker Daltonics) mass spectrometer using Peptide Mass Standard II (Bruker Daltonics) as calibrant. The five most intense mass spectrometry signals were selected for tandem mass spectrometry analysis. MASCOT (version 2.1.02; Matrix Science, London, UK) searching against protein predictions from the *A. benhamiae *genome and the NCBI database (taxa fungi) was used for protein identification with the following the parameters: fixed modification of cysteine to S-carbamidomethyl derivatives, variable methionine oxidation, no missed cleavage and a peptide mass tolerance of 200 ppm.

### PKS and NRPS domain architecture prediction

The PKS and NRPS domain architecture was predicted using the InterProScan [55] and NRPS-PKS [56] tools.

### Generation of the phylogenetic tree

For genome-based phylogeny, 23 proteins from 28 fully sequenced fungal genomes were used for the reconstruction of the phylogenetic relationships of *A. benhamiae *and *T. verrucosum *(Additional file 3). The 23 ortholog groups were selected based on the KOG (clusters of orthologous groups for eukaryotes) assignments, as described by Xu *et al*. [23]. Only KOGs without paralogs, that is, proteins represented by a single protein in a species, were taken into consideration. Five proteins from the publication of Xu *et al*. [23] were not confirmed as fulfilling this requirement. Thus, they were not included. The genome set selected for the survey was non-redundant, that is, we did not consider four closely related *Candida *species as well as six *Saccharomyces *species, but only representatives of each clade, that is, *C. albicans *and *S. cerevisiae*, respectively. By contrast, we included all available Pezizomycetes, since *A. benhamiae *and *T. verrucosum *presumably belong to this phylum. A representative of Zygomycota (*Rhizopus oryzae*) was used as an outgroup. The considered genomes were as follws. Eurotiomycetes: *Arthroderma benhamiae*, *Trichophyton verrucosum*, *Aspergillus clavatus*, *Aspergillus flavus*, *Aspergillus fumigatus*, *Aspergillus nidulans*, *Aspergillus oryzae*, *Aspergillus terreus*, *Botrytis cinerea*, *Coccidioides immitis*, *Histoplasma capsulatum*, *Paracoccidioides brasiliensis*, *Sclerotinia sclerotiorum*, *Stagonospora nodorum*, *Uncinocarpus reesii*. Sordariomycetes: *Chaetomium globosum*, *Fusarium graminearum*, *Magnaporthe grisea*, *Neurospora crassa*. Saccharomycotina: *Candida albicans*, *Lodderomyces elongisporus*, *Saccharomyces cerevisiae*. Taphrinomycotina: *Schizosaccharomyces japonicus*. Basidiomycota: *Coprinus cinereus*, *Cryptococcus neoformans*, *Puccinia graminis*, *Ustilago maydis*. Zygomycota: *Rhizopus oryzae*.

The protein sets for each KOG protein shared among the 28 genomes were collected. Each set was then aligned by ClustalX, and the conserved blocks were extracted using the Gblocks tool [57] with allowance of smaller final blocks (five amino acids) and gap positions within the final blocks using otherwise default parameters. The extracted blocks were concatenated for each species. The phylogenetic analysis was performed using PHYML [58] for the construction of the maximal likelihood tree, and PHYLIP for the construction the neighbor joining tree, with the Jones-Taylor-Thornton model of the amino acid substitution in both cases. The neighbor joining and maximal likelihood trees had identical architecture.

The phylogenetic trees for proteases and enzymes involved in SM production were obtained using PHYLIP for the construction of the neighbor joining tree, with the Jones-Taylor-Thornton model of the amino acid substitution.

## Abbreviations

ACP: acyl carrier protein domain; AT: acetyltransferase domain; DMEM: Dulbecco's Modified Eagle's medium; DTT: dithiothreitol; EST: expressed sequence tag; KR: ketoacyl reductase domain; KS: beta-ketoacyl synthase domain; Lap: leucine aminopeptidase; MAT locus: mating type locus; ME: methyltransferase domain; Mep: metalloprotease, fungalysin; NRPS: non-ribosomal peptide synthetase; PKS: polyketide synthase; SG medium: Sabouraud 2% glucose medium; SM: secondary metabolite; Sub: subtilisin-like protease.

## Authors' contributions

AAB initiated the study; AAB, JW, CH, MP, PS, PFZ, and RG designed the research; AB prepared DNA and fosmid libraries and carried out mating type analysis; ES, MF, GG, RG, WL, and SP carried out bioinformatic analyses; GG, AH, KS, MF, AP, MP, ES, MG, and VS performed genome and transcriptome sequence analysis; CHed, RW, and OK carried out proteome analysis; SS, CHed and PFZ performed experiments with human keratinocytes; MM provided fungal materials and critical discussions; MM, MFeu and IP performed analysis of proteases; AAB, PS, ES, MM, BH, CH, JH, and PFZ analyzed the results; TCW participated in the design and coordination of research and provided critical discussions; PS, ES, CHed, and AAB wrote the paper. All authors read and approved the final manuscript.

## Supplementary Material

Additional file 1 **Supplementary information to Figures **1**and **3. Click here for file

Additional file 2 **Supplementary data on genome structure of dermatophytes**. Table S1a: a detailed description of the sequencing. Table S1b: information on combined assembly. Figure S1: found translocations and the inverson. Click here for file

Additional file 3 **Generation of the phylogenetic tree**. The file contains the whole phylogenetic tree (Figure S2) and a table of genes used for its construction. Click here for file

Additional file 4 **Species-specific genes**. The Excel file contains lists of genes that do not have counterparts in the other genome. Click here for file

Additional file 5 **Table S2: Fast-evolving *A. benhamiae *genes (dN/dS >1)**. Click here for file

Additional file 6 **supplementary Tables S3, S5, S6, S7, S8, and S9**. Table S3: predicted proteases with marked proteases with secretion signal according to SignalP predictions. Table S5: identification and prediction of secretion signals of protein spots shown in Figure 3. Table S6: comparison of dermatophyte secretome data of Giddey *et al*. [4 ] and the present study. Table S7: differentially expressed genes of *A. benhamiae *during co-cultivation with human keratinocytes. Table S8: genes implicated in sexual reproduction and meiosis-specific genes. Table S9: numbers of reads obtained in the transcriptome analysis of infection and control samples. Click here for file

Additional file 7 **Table S4: secreted proteases in *A. benhamiae*, *T. verrucosum*, *Aspergillus fumigatus *and *Coccidioides *spp**. Click here for file

Additional file 8 **Phylogenetic trees of secreted proteases**. The file contains the phylogenies of the *A. benhamiae*, *T. verrucosum*, and *Coccidioides *secreted proteases of the most distinguishing families S8, M35, and M36 (Figure S3.1, S3.2, and S3.3, respectively). Click here for file

Additional file 9 **Figure S4: Northern Blot analysis**. Click here for file

Additional file 10 **Phylogenetic trees of *A. benhamiae*, *T. verrucosum*, and *Coccidioides immitis *PKSs and NRPSs**. The file contains phylogenetic trees built for NRPSs (Figure S5.1) and PKSs (Figure S5.2), comparing the corresponding genes sets of the three species. Click here for file
