# Supplementary material for: Comparative and functional genomics provide insights into the pathogenicity of dermatophytic fungi
Source: Genome Biol. 2011 Jan 19;12(1):R7. doi: 10.1186/gb-2011-12-1-r7 (PMC3091305; doi:10.1186/gb-2011-12-1-r7)
Supplement: Additional file 1 — Supplementary information to Figures 1and 3. [file gb-2011-12-1-r7-S1.DOC]

**Supplementary information to Figs. 1 and 3**

**Figs. 1A and 3A:** 10^3^ microconidia of *A. benhamiae* were added to a 1 % (w/v) keratin suspension in 10 µl and cultivated on cover slides in a humid chamber for 10 days at 30 °C. Hyphae were stained with 10 µg/ml Fluorescent Brightener (FB) 28 (Sigma). Image stacks were obtained using the Zeiss LSM 5 *LIVE* laser scanning microscope with ZEN 2007 software. One channel was used to detect the FB 28 fluorescense signal, the second for the keratin particle autofluorescence (GFP – filter). The 3D render mode was ‘Maximum’ for Fig.1a and ‘Shadow’ for Fig. 3a.

**Fig. 1B:** 10^6^ microconidia were streaked onto 1.5 % (w/v) agar plates. Sterilized hair was added and incubated for 14 days at 30 °C. Microscopy was as described for images 1a and 3a using FB 28 for hyphal staining, the hair autofluorescence signal and ‘Shadow’ rendering for the 3D view.

**Fig. 1C:** Microconidia were added to keratinocytes (HaCaT) and incubated for 180 min in DPBS. After washing with DPBS, cells were fixed in 3% (v/v) p-formaldehyde for 15 min. Then, keratinocytes and *A. benhamiae* microconidia were stained for 10 min with 4 µg/ml wheat-germ agglutinin and with 1 µg/ml FB 28, respectively. All steps were performed at room temperature. Between each staining step, cells were washed twice with DPBS. An Olympus BX51 fluorescense microscope was used to obtain the image.

**Fig. 1D:** *A. benhamiae* microconidia were co-incubated with keratinocytes for 3 hours at room temperature in DPBS. Cells were washed twice with 0.1 M cacodylate buffer pH 7.2 and fixed with 2.5 % (v/v) glutaraldehyde in cacodylate buffer at room temperature for 45 min. After washing with 0.1 M cacodylate buffer, cells where dehydrated using an increasing ethanol series followed by critical point drying and gold sputtering. For microscopy, LEO-1450 VP scanning electron microscope was used.
