# Supplementary material for: Comparative and functional genomics provide insights into the pathogenicity of dermatophytic fungi
Source: Genome Biol. 2011 Jan 19;12(1):R7. doi: 10.1186/gb-2011-12-1-r7 (PMC3091305; doi:10.1186/gb-2011-12-1-r7)
Supplement: Additional file 2 — Supplementary data on genome structure of dermatophytes. Table S1a: a detailed description of the sequencing. Table S1b: information on combined assembly. Figure S1: found translocations and the inverson. [file gb-2011-12-1-r7-S2.DOC]

**Supplementary data on genome structure of dermatophytes**

For *A. benhamiae* 30,720 plasmid clones were sequenced from both ends. We produced 59,333 individual good quality sequences, which covered the genome approximately 2 times. Furthermore, 4,320 fosmid clones were sequenced from both ends resulting in 7,550 good quality sequences and a clone coverage of approximately 7 times. For *T. verrucosum*, almost 2,000 fosmid clones were end sequenced, providing 3,344 good quality sequences and an estimated clone coverage of 3 times. The final individual assemblies of both species comprised 68 and 523 contigs, respectively, yielding approximately the same length for both genomes with 22.5 Mb (Table 1). Many contigs in the *A. benhamiae* genome are bordered by sequences, which deviate strikingly from the mean GC content of 48 %. The DNA sequence in these regions is information poor (monotone) which presumably is the reason for the failure to resolve these regions using the short next generation sequencing reads. Surprisingly, we found no fosmid clones spanning the gaps at A/T rich sequences in the *A. benhamiae* genome, indicating a cloning bias against A/T rich sequences or the end of a chromosome at current gap positions. These sequences apparently do not code for protein coding genes. Therefore, they are most likely not transposons of known types. These sequences can be longer than 10 kb, as some non-coding contigs comprised mainly of Sanger sequencing reads demonstrate. However, the nature and function of these interspersed sequences is not clear. They sometimes have some sequence similarity to each other, which also hinders their proper assembly from short 454 reads. Due to this similarity we can conclude that they possibly have the same origin or are generated by the same mechanism. It remains to be determine, how these sequences are generated and inserted in the genome.

Furthermore, eleven entire contigs comprising together 58 kb of sequence have an average G/C content of considerably less than 45 %. Three of these contigs contain five genes altogether, the other contigs do not code for any detectable gene. These contigs are probably located in gaps bordered by A/T rich sequences. Taken together, the *A. benhamiae* genome seems to represent a mosaic of long GC rich, gene-containing portions separated by AT rich “islands”, which could reach lengths of more than 10 kb, possibly causing a cloning bias against these regions.

A comparison of the *T. verrucosum* genome with that of *A. benhamiae* shows that the two species are very closely related. Polymorphisms and insertions or deletions (indels) between the two sequences are generally scarce. Using the softeware Mummer [21] ~21.8 Mb of the genomes can be aligned to each other indicating that the majority of all genes lays in syntenic regions and is shared between the two organisms. The average identity of the alignable portion of the genomes is 94.8 % but in some regions we observed greater diversity or indels. An exception in the overall high similarity are also the A/T rich island sequences. These genomic regions fail to be similar to their counterparts. Moreover, not all positions of these A/T rich islands are the same in the two genomes accounting for the majority of alignment breaks. Assuming that most regions of the two genomes are collinear, we used the complete genome of *A. benhamiae* to bridge the gaps between the contigs of *T. verrucosum*. Using this approach, we were able to bridge 397 gaps in the *T. verrucosum* genome (Supplemental Table A1b). A subset of 15 gaps was tested by designing primers for PCR spanning the gaps in *T. verrucosum*, and all 15 primer pairs produced PCR products of the expected length. Considering that in the selection of the gaps for testing, 3 were skipped due to the difficulty to design appropriate primers or longer gaps, then conservatively we can extrapolate that at least 80% of the gaps are correctly closed. The true percentage is possibly higher since the not closed gaps are likely real, but more difficult to close. Furthermore, this global alignment revealed that *T. verrucosum* has probably 36 A/T island insertions in this consensus sequence of both species. The merged assembly consists of more contigs than that of *A. benhamiae* mainly due to these 36 A/T rich interspersed sequences of *T. verrucosum*. The remaining three additional contigs in the merged assembly are A/T rich and are probably located in gaps between contigs bordered by A/T rich sequences. We also could define 5 reciprocal translocations and one inversion of 77.5 kb between the two genomes (Supplemental Figure A1). Since most gaps in the coding regions of *A. benhamiae* were closed and only gaps in A/T rich regions remained, these numbers should reflect very closely the overall genomic structural diversity of the two species. Since the *T. verrucosum* genome is too fragmented, we cannot determine the number and location of A/T island insertions in the *A. benhamiae* genome. However, the observed number of remaining gaps, which are due to A/T rich sequences, indicates that the number of A/T rich insertions should be in the same range as for *T. verrucosum*.

**Supplemental table S1.** Sequence and assembly of genomes

| S1a. Sequence and assembly of genomes | | | | |
| --- | --- | --- | --- | --- |
|  | | | ***A. benhamiae*** | ***T. verrucosum*** |
| **Raw**  **data** | 454/FLX (Mb) | | 424 | 407 |
|  | Sanger fosmid sequences | | 7580 | 3344 |
|  | Sanger pUC sequences | | 59153 | 180 |
| **454**  **assembly** | Contigs | | 853 | 849 |
|  | Nucleotides (Mb) | | 22.3 | 23 |
|  | Average length (kb) | | 26.2 | 27.1 |
|  | Largest contig (kb) | | 313.2 | 265.9 |
|  | N50 contig length (kb) | | 60.5 | 73.2 |
| **Hybrid**  **assembly**  **(contigs**  **>3.5 kb)** | Contigs | | 69* | 524* |
|  | Nucleotides (Mb) | | 22.3 | 22.6 |
|  | Average contig length (kb) | | 322.5 | 43.1 |
|  | Largest contig (Mb) | | 2.1 | 0.27 |
| S1b. Summary information on the combined assembly | | | | |
| Contigs | | 126 | | |
| Total contig length (bp) | | 22944216 | | |
| Largest contig (bp) | | 1873278 | | |
| Gaps closed | | 397 | | |

* + mitchondrial genome


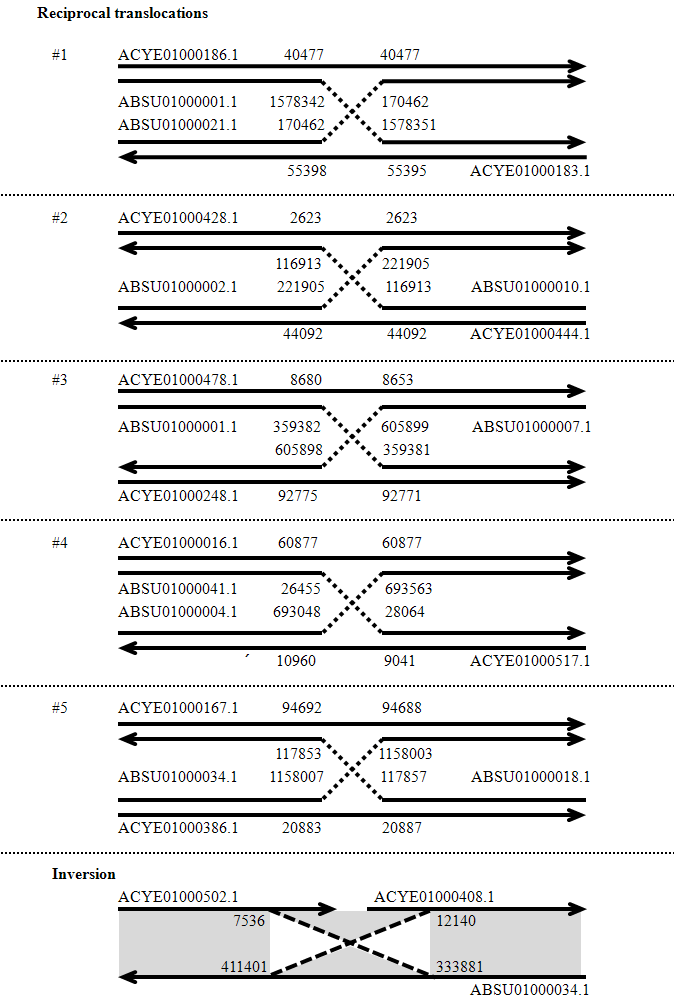


**Supplemental figure S1.** Reciprocal translocations and an inversion between the two genomes: GenBank accession numbers are given as identifiers for all contigs, and arrows indicate the direction of the forward strand in the GenBank file. Reciprocal translocations: *T. verrucosum* contigs are on the outside with *A. benhamiae* contigs in between. Numbers indicate the position in the contig where the translocation occurs. Dashed lines indicate where an *A. benhamiae* contig switches from homology to one *T. verrucosum* contig to another (i.e., in the first translocation, *T. verrucosum* contig ACYE01000186.1 upstream of position 40477 is similar to *A. benhamiae* contig ABSU01000001.1, and downstream of this point, there is similarity to *A. benhamiae* contig ABSU01000021.1). Positions are derived from Nucmer alignments and may appear to overlap, if there are common bases on both sides of the break point because then the exact break point can not be determined. Inversion: The inversion is spread between two contigs in *T. verrucosum* but is contained in a single *A. benhamiae* contig. Dashed lines indicate homology between the sequences (i.e., in contig ACYE01000502.1 upstream of position 7536 there is homology to sequence downstream of position 411401 in contig ABSU01000034.1).
