# Supplementary material for: Comparative and functional genomics provide insights into the pathogenicity of dermatophytic fungi
Source: Genome Biol. 2011 Jan 19;12(1):R7. doi: 10.1186/gb-2011-12-1-r7 (PMC3091305; doi:10.1186/gb-2011-12-1-r7)
Supplement: Additional file 3 — Generation of the phylogenetic tree. The file contains the whole phylogenetic tree (Figure S2) and a table of genes used for its construction. [file gb-2011-12-1-r7-S3.DOC]

## Generation of the phylogentic tree

##

Fig. S2. Genome-based phylogeny of *A. benhamiae* and *T. verrucosum.*

##### List of proteins used for phylogenetic analysis

| **KOG number** | **KOG functional category** | **Protein name/ function** |
| --- | --- | --- |
| KOG0325 | Energy production and conversion [C],  Coenzyme transport and metabolism [H] | Lipoyltransferase |
| KOG0436 | Translation [J] | Methionyl-tRNA synthetase |
| KOG0809 | Intracellular trafficking and secretion [U] | SNARE protein TLG2/syntaxin 16 |
| KOG0816 | RNA processing and modification [A] | Protein involved in mRNA turnover |
| KOG0991 | Replication, recombination and repair [L] | DNA replication factor C subunit Rfc2 |
| KOG1173 | Cell cycle control, mitosis and meiosis [D],  Posttranslational modification, protein turnover, chaperones [O] | Anaphase-promoting complex (APC), Cdc16 subunit |
| KOG1534 | RNA processing and modification [A] ,  Transcription [K] | Transcription factor FET5 |
| KOG1598 | Transcription [K] | transcription factor TFIIIB complex subunit Brf1 |
| KOG1750 | Translation [J] | ribosomal protein S23 (S12) |
| KOG2267 | Replication, recombination and repair [L] | DNA primase large subunit Pri2 |
| KOG2626 | Transcription [K],  Chromatin structure and dynamics [B] | Histone H3 (lys4) methyltransferase complex, subunit CPS60/AHS2/BRE2 |
| KOG2671 | Replication, recombination and repair [L] | RNA methylase family protein |
| KOG2728 | General function prediction only [R] | Uncharacterized conserved protein with similarity to phosphopantothenoylcysteine synthase/decarboxylase |
| KOG2794 | Coenzyme transport and metabolism [H] | Delta-aminolevulinic acid dehydratase |
| KOG2851 | Replication, recombination and repair [L] | DNA primase small (catalytic) subunit Pri1 |
| KOG2874 | Translation [J],  Cell cycle control, mitosis and meiosis [D] | rRNA processing protein |
| KOG3013 | RNA processing and modification [A] | exosomal 3’-5’ exoribonuclease complex Rrp4 |
| KOG3104 | Transcription [K] | Mod5 protein sorting/negative effector of RNA PolIII synthesis |
| KOG3185 | Translation [J] | translation initiation factor eIF-6 |
| KOG3327 | Nucleotide transport and metabolism [F] | thymidylate kinase |
| KOG3789 | Inorganic ion transport and metabolism [P] | nitrogen permease regulator NLRG/Npr2 |
| KOG3800 | Posttranslational modification, protein turnover, chaperones [O] | E3 RING finger ubiquitin ligase (Tul1) |
