# Supplementary material for: Comparative and functional genomics provide insights into the pathogenicity of dermatophytic fungi
Source: Genome Biol. 2011 Jan 19;12(1):R7. doi: 10.1186/gb-2011-12-1-r7 (PMC3091305; doi:10.1186/gb-2011-12-1-r7)
Supplement: Additional file 5 — Table S2: Fast-evolving A. benhamiae genes (dN/dS >1). [file gb-2011-12-1-r7-S5.DOC]

**Table S2.** Fast-evolving *A. benhamiae* genes (dN/dS > 1)

| Reference | name | dN/dS |
| --- | --- | --- |
| NA/ARB_05625 | PXA domain protein | 3.88 |
| NA/ARB_02249 | TCTP family protein | 3.50 |
| NA/ARB_06931 | Woronin body protein HexA, putative | 2.74 |
| NA/ARB_03304 | integral membrane protein, Mpv17/PMP22 family, putative | 2.40 |
| NA/ARB_07923 | mitochondrial import protein Zim17, putative | 2.36 |
| NA/ARB_05893 | C6 transcription factor, putative | 2.23 |
| NA/ARB_01197 | peptidyl-tRNA hydrolase domain protein | 2.23 |
| NA/ARB_05233 | RNA polymerase II transcription factor SIII (Elongin) subunit A, putative | 2.20 |
| NA/ARB_07547 | 26S proteasome non-ATPase regulatory subunit Nas2, putative | 2.13 |
| NA/ARB_02078 | aminotransferase, putative | 2.09 |
| NA/ARB_03674 | cytosolic Cu/Zn superoxide dismutase, putative | 2.04 |
| NA/ARB_07160 | NmrA-like family protein, putative | 2.04 |
| NA/ARB_07412 | ankyrin repeat protein | 2.00 |
| NA/ARB_00531 | C6 transcription factor (OTam), putative | 2.00 |
| NA/ARB_06263 | DNA damage response protein RcaA | 2.00 |
| NA/ARB_05919 | lysophospholipase Plb2 | 2.00 |
| NA/ARB_02722 | oxidoreductase, short chain dehydrogenase/reductase family | 2.00 |
| NA/ARB_01001 | proteasome component (Ecm29), putative | 2.00 |
| NA/ARB_03765 | proteinase, putative | 2.00 |
| NA/ARB_02559 | ubiquitin C-terminal hydrolase 37 | 2.00 |
| NA/ARB_03206 | integral membrane protein, putative | 1.95 |
| NA/ARB_00548 | cytochrome P450 monooxygenase, putative | 1.88 |
| NA/ARB_01131 | cytochrome P450 alkane hydroxylase, putative | 1.85 |
| NA/ARB_06027 | replication fork protection component Swi3, putative | 1.83 |
| NA/ARB_03684 | bZIP transcription factor CpcA | 1.81 |
| NA/ARB_01501 | bifunctional catalase-peroxidase Cat2 | 1.76 |
| NA/ARB_05304 | allergenic cerato-platanin Asp F13 | 1.75 |
| NA/ARB_02519 | C2H2 finger domain protein, putative | 1.75 |
| NA/ARB_03861 | glutathione S-transferase Ure2-like, putative | 1.75 |
| NA/ARB_03966 | serine/threonine protein kinase, putative | 1.75 |
| NA/ARB_04758 | mitochondrial carrier protein, putative | 1.74 |
| NA/ARB_04643 | NmrA-like family protein | 1.72 |
| NA/ARB_04861 | peptidase family M20/M25/M40 protein | 1.72 |
| NA/ARB_06887 | DnaJ domain protein | 1.71 |
| NA/ARB_01456 | MAP kinase MpkC | 1.64 |
| NA/ARB_01661 | nuclear RNA binding protein, putative | 1.63 |
| NA/ARB_05249 | oxidoreductase, zinc-binding dehydrogenase family, putative | 1.63 |
| NA/ARB_00554 | serine/threonine protein kinase, putative | 1.63 |
| NA/ARB_01984 | thioesterase family protein | 1.63 |
| NA/ARB_01771 | vacuolar ATPase proteolipid subunit c, putative | 1.63 |
| NA/ARB_01794 | Ctr copper transporter, putative | 1.62 |
| NA/ARB_03691 | HMG box protein, putative | 1.61 |
| NA/ARB_02172 | bZIP transcription factor (LziP), putative | 1.59 |
| NA/ARB_05223 | C2H2 transcription factor (Con7), putative | 1.59 |
| NA/ARB_03299 | PT repeat family protein | 1.52 |
| NA/ARB_06662 | SET domain protein | 1.52 |
| NA/ARB_07318 | DNA lyase Apn2 | 1.51 |
| NA/ARB_01419 | Acyl CoA binding protein family | 1.50 |
| NA/ARB_05027 | ankyrin repeat protein | 1.50 |
| NA/ARB_00648 | class V chitinase Chi100 | 1.50 |
| NA/ARB_01014 | glucokinase regulator family protein, putative | 1.50 |
| NA/ARB_00955 | G-patch RNA maturation protein (Gno1), putative | 1.50 |
| NA/ARB_00969 | small nuclear ribonucleoprotein SmF, putative | 1.50 |
| NA/ARB_03395 | solid-state culture expressed protein (Aos23), putative | 1.50 |
| NA/ARB_02997 | peptidase S41 family protein | 1.48 |
| NA/ARB_03118 | acetyltransferase, GNAT family family | 1.44 |
| NA/ARB_06022 | C6 transcription factor, putative | 1.44 |
| NA/ARB_00205 | cell cycle inhibitor Nif1, putative | 1.44 |
| NA/ARB_01017 | CFEM domain protein, putative | 1.43 |
| NA/ARB_05195 | conserved glutamic acid-rich protein | 1.43 |
| NA/ARB_00326 | PEX11 domain protein | 1.43 |
| NA/ARB_05719 | RNA polymerase II Elongator complex associated protein Kti12, putative | 1.43 |
| NA/ARB_01657 | MFS transporter, putative | 1.42 |
| NA/ARB_01805 | oxidoreductase, short-chain dehydrogenase/reductase family | 1.42 |
| NA/ARB_07486 | SprT family metallopeptidase, putative | 1.42 |
| NA/ARB_03377 | cell morphogenesis protein Las1, putative | 1.41 |
| NA/ARB_02417 | telomerase reverse transcriptase, putative | 1.41 |
| NA/ARB_02123 | conserved glutamic acid-rich protein | 1.40 |
| NA/ARB_03375 | mitochondrial inner membrane protease subunit 1, putative | 1.40 |
| NA/ARB_00257 | ribonucleoprotein, putative | 1.40 |
| NA/ARB_00468 | C2H2 transcription factor, putative | 1.39 |
| NA/ARB_05564 | MFS multidrug transporter, putative | 1.39 |
| NA/ARB_06564 | Myb-like DNA-binding domain protein | 1.38 |
| NA/ARB_02221 | transferase family protein | 1.36 |
| NA/ARB_02373 | DNA repair protein Pso2/Snm1, putative | 1.35 |
| NA/ARB_06932 | MFS transporter, putative | 1.35 |
| NA/ARB_04736 | F-box domain and ankyrin repeat protein | 1.34 |
| NA/ARB_06663 | long-chain-fatty-acid-CoA ligase, putative | 1.34 |
| NA/ARB_02201 | C6 finger domain protein, putative | 1.33 |
| NA/ARB_01606 | integral ER membrane protein Scs2, putative | 1.33 |
| NA/ARB_03871 | integral membrane protein | 1.33 |
| NA/ARB_07177 | MFS transporter, putative | 1.33 |
| NA/ARB_04277 | transferase (Gpi7), putative | 1.32 |
| NA/ARB_04288 | vegetative incompatibility WD repeat protein, putative | 1.31 |
| NA/ARB_04720 | WD repeat protein | 1.30 |
| NA/ARB_04810 | DnaJ domain protein | 1.29 |
| NA/ARB_04747 | SUN domain protein (Uth1), putative | 1.29 |
| NA/ARB_05647 | tRNA-specific adenosine deaminase, putative | 1.29 |
| NA/ARB_01140 | 1,3-beta-glucanosyltransferase, putative | 1.27 |
| NA/ARB_02034 | GNAT family acetyltransferase, putative | 1.27 |
| NA/ARB_02583 | bZIP transcription factor JlbA/IDI-4 | 1.25 |
| NA/ARB_03577 | class V chitinase, putative | 1.25 |
| NA/ARB_00099 | ribonuclease H1, putative | 1.25 |
| NA/ARB_04195 | RING finger domain protein (Rnf10), putative | 1.25 |
| NA/ARB_07206 | TBP interacting domain protein, putative | 1.25 |
| NA/ARB_06076 | alkaline serine protease (PR1)/allergen F18-like | 1.24 |
| NA/ARB_07716 | involucrin repeat protein ( | 1.24 |
| NA/ARB_00303 | MIZ zinc finger domain protein | 1.24 |
| NA/ARB_01353 | beta-N-hexosaminidase, putative | 1.23 |
| NA/ARB_01788 | homeobox transcription factor, putative | 1.23 |
| NA/ARB_03273 | PHD finger domain protein, putative | 1.22 |
| NA/ARB_04688 | tRNA m(1)G methyltransferase domain containing protein | 1.21 |
| NA/ARB_02555 | C2H2 finger domain protein, putative | 1.20 |
| NA/ARB_06921 | urea amidolyase, putative | 1.20 |
| NA/ARB_07310 | anaphase-promoting complex subunit Apc5, putative | 1.18 |
| NA/ARB_03180 | DNA polymerase POL4, putative | 1.18 |
| NA/ARB_03211 | C6 and C2H2 transcription factor RegA-like, putative | 1.17 |
| NA/ARB_02369 | carboxylesterase, putative | 1.17 |
| NA/ARB_04408 | MYND domain protein (SamB), putative | 1.15 |
| NA/ARB_01270 | ankyrin repeat protein | 1.14 |
| NA/ARB_05864 | cell wall glucanase (Scw11), putative | 1.14 |
| NA/ARB_07698 | MOSC domain protein | 1.14 |
| NA/ARB_02298 | SET domain protein | 1.14 |
| NA/ARB_02797 | GPI-anchored cell wall beta-1,3-endoglucanase EglC | 1.13 |
| NA/ARB_01321 | C6 finger domain protein, putative | 1.12 |
| NA/ARB_07948 | FAD-binding oxidoreductase, putative | 1.12 |
| NA/ARB_00597 | integral membrane protein Pth11-like, putative | 1.10 |
| NA/ARB_00022 | mitochondrial export translocase Oxa2, putative | 1.10 |
| NA/ARB_02904 | MFS multidrug transporter, putative | 1.09 |
| NA/ARB_06280 | Protein kinase domain-containing protein | 1.08 |
| NA/ARB_04583 | Leucine Rich Repeat domain protein | 1.06 |
| NA/ARB_04502 | proline oxidase PrnD | 1.06 |
| NA/ARB_01681 | CHL4 family chromosome segregation protein, putative | 1.05 |
| NA/ARB_07542 | RanBP1 domain protein | 1.05 |
| NA/ARB_02603 | shugoshin family protein | 1.05 |
| NA/ARB_05929 | aldehyde dehydrogenase ALDH | 1.04 |
| NA/ARB_05565 | C6 finger domain protein, putative | 1.04 |
| NA/ARB_00943 | C6 finger domain protein, putative | 1.03 |
| NA/ARB_01691 | DNA mismatch repair protein (Mlh3), putative | 1.03 |
| NA/ARB_02629 | DNA polymerase epsilon subunit B, putative | 1.02 |
| NA/ARB_05346 | nuclear membrane fusion protein Kar5, putative | 1.02 |
| NA/ARB_00226 | raffinose synthase protein Sip1, putative | 1.02 |
