# Supplementary material for: Comparative and functional genomics provide insights into the pathogenicity of dermatophytic fungi
Source: Genome Biol. 2011 Jan 19;12(1):R7. doi: 10.1186/gb-2011-12-1-r7 (PMC3091305; doi:10.1186/gb-2011-12-1-r7)
Supplement: Additional file 6 — supplementary Tables S3, S5, S6, S7, S8, and S9. Table S3: predicted proteases with marked proteases with secretion signal according to SignalP predictions. Table S5: identification and prediction of secretion signals of protein spots shown in Figure 3. Table S6: comparison of dermatophyte secretome data of Giddey et al. [4 ] and the present study. Table S7: differentially expressed genes of A. benhamiae during co-cultivation with human keratinocytes. Table S8: genes implicated in sexual reproduction and meiosis-specific genes. Table S9: numbers of reads obtained in the transcriptome analysis of infection and control samples. [file gb-2011-12-1-r7-S6.DOC]

# Additional tables S3, S5-S9

**Table S3.** List of predicted proteases.^s^ – secretion signal according to SignalP predictions

| **Locuslink** | **Name/description** | **MEROPS hit** |
| --- | --- | --- |
| ARB_01612  ^s^ | Serine/cysteine peptidase, trypsin-like |  |
| ARB_01885  ^s^ | Serine/cysteine peptidase, trypsin-like | S64 |
| ARB_02186  ^s^ | metallopeptidase |  |
| ARB_06229  ^s^ | Beta-lactamase-type transpeptidase fold | S12 |
| ARB_07764 | Peptidase, cysteine peptidase active site |  |
| ARB_00657 | Serine/cysteine peptidase, trypsin-like |  |
| ARB_00717 | Serine/cysteine peptidase, trypsin-like |  |
| ARB_00596 | serine/cysteine peptidase, trypsin-like |  |
| ARB_05025 | hypothetical protein, Serine/cysteine peptidase, trypsin-like | S01X |
| ARB_01468 | conserved hypothetical protein, Peptidase S8/S53, subtilisin/kexin/sedolisin |  |
| ARB_02724 | Serine/cysteine peptidase, trypsin-like |  |
| ARB_07539 | Peptidase S10, serine carboxypeptidase, active site, Fungal specific transcription factor |  |
| ARB_00043 ^s^ | Peptidase C13, legumain | C13 |
| ARB_00083 ^s^ | serine peptidase, family S28, putative | S28 |
| ARB_00141 ^s^ | ubiquitin C-terminal hydrolase CreB | C19 |
| ARB_00230 ^s^ | Peptidase M28 | M28X |
| ARB_00506 ^s^ | aminopeptidase | M18 |
| ARB_00576 ^s^ | Peptidase M28 | M28E |
| ARB_00683 ^s^ | ubiquitin C-terminal hydrolase, putative | C19 |
| ARB_00701 ^s^ | subtilisin-like protease SUB3 | S08A |
| ARB_00762 ^s^ | fungalysin/metallopeptidase | M36,M04 |
| ARB_00777 ^s^ | proteinase | S08A |
| ARB_00812 ^s^ | Proteasome, subunit alpha/beta | T01A |
| ARB_00849 ^s^ | penicillolysin/deuterolysin metalloprotease, putative | M35 |
| ARB_00890 ^s^ | Gamma-glutamyltranspeptidase | T03 |
| ARB_00930 ^s^ | beta-lactamase, putative | S12 |
| ARB_01032 ^s^ | subtilisin-like protease SUB4 | S08A |
| ARB_01041 ^s^ | peptidase, putative | M20A |
| ARB_01287 ^s^ | separin, putative Peptidase C50, separase | C50 |
| ARB_01307 ^s^ | Peptidase, cysteine peptidase active site; Protein kinase |  |
| ARB_01311 ^s^ | Proteasome, subunit alpha/beta | T01A |
| ARB_01345 ^s^ | extracelular serine carboxypeptidase, putative | S28 |
| ARB_01382 ^s^ | fungalysin/metallopeptidase | M04,M36 |
| ARB_01443 ^s^ | Peptidase M28 | M28E |
| ARB_01491 ^s^ | carboxypeptidase S1, putative | S10 |
| ARB_01495 ^s^ | subtilisin-like protease SUB2 | S08A |
| ARB_01953 ^s^ | Peptidase C19, ubiquitin carboxyl-terminal hydrolase 2 | C19 |
| ARB_02007 ^s^ | signal peptide peptidase, putative | A22B |
| ARB_02099 ^s^ | aspartic-type endopeptidase, putative |  |
| ARB_02223 ^s^ | subtilisin-like protease SUB5 | S08A |
| ARB_02289 ^s^ | ADAM family of metalloprotease ADM-A | M12B |
| ARB_02296 ^s^ | Peptidase S59, nucleoporin | S08A,S59 |
| ARB_02406 ^s^ | fungalysin/metallopeptidase | M04,M36 |
| ARB_02407 ^s^ | Peptidase M14, carboxypeptidase A | M14X |
| ARB_02559 ^s^ | ubiquitin C-terminal hydrolase 37 | C12 |
| ARB_02670 ^s^ | dipeptidyl peptidase III | M49 |
| ARB_02715 ^s^ | Peptidase M19, renal dipeptidase | M19 |
| ARB_02919 ^s^ | Peptidase A1 | A01A |
| ARB_02921 ^s^ | Gamma-glutamyltranspeptidase | T03 |
| ARB_02997 ^s^ | peptidase S41 family protein |  |
| ARB_03419 ^s^ | Peptidase aspartic | M38 |
| ARB_03476 ^s^ | ubiquitin C-terminal hydrolase, putative | C19 |
| ARB_03492 ^s^ | Peptidase M28 | M28E |
| ARB_03758 ^s^ | Peptidase S10 | S10 |
| ARB_03820 ^s^ | Peptidase S26A, signal peptidase I, conserved site, Alpha/beta hydrolase fold-1 | S33 |
| ARB_03949 ^s^ | metalloproteinase, putative | M35 |
| ARB_04018 ^s^ | Peptidase A1 | A01A |
| ARB_04046 ^s^ | carboxypeptidase S1, putative | S10 |
| ARB_04101 ^s^ | serine protease, putative | S53 |
| ARB_04153 ^s^ | Peptidase M22 | M22 |
| ARB_04170 ^s^ | aspartic-type endopeptidase (OpsB), putative | A01A |
| ARB_04261 ^s^ | aminopeptidase | M24B |
| ARB_04341 ^s^ | Peptidase M16 | M16B |
| ARB_04447 ^s^ | Peptidase M22, glycoprotease | M22 |
| ARB_04677 ^s^ | proteinase |  |
| ARB_04769 ^s^ | metallopeptidase | M35 |
| ARB_04807 ^s^ | Peptidase S10, serine carboxypeptidase | S10 |
| ARB_04985 ^s^ | Beta-lactamase-type transpeptidase fold | S12 |
| ARB_05085 ^s^ | fungalysin/metallopeptidase | M04,M36 |
| ARB_05307 ^s^ | subtilisin-like protease SUB6 | S08A |
| ARB_05317 ^s^ | metallopeptidase | M43B |
| ARB_05721 ^s^ | carboxypeptidase Y, putative | S10 |
| ARB_05728 ^s^ | aspartic endopeptidase (AP1), putative | A01A |
| ARB_05765 ^s^ | tripeptidyl-peptidase (TppA), putative | S53 |
| ARB_05817 ^s^ | metallopeptidase | M35 |
| ARB_06019 ^s^ | carboxypeptidase S1, putative | S10 |
| ARB_06110 ^s^ | Peptidase S9 | S09B |
| ARB_06414 ^s^ | pheromone processing carboxypeptidase (Sxa2), putative | S10 |
| ARB_06416 ^s^ | proteinase | S08A |
| ARB_06467 ^s^ | proteinase | S08A |
| ARB_06472 ^s^ | metalloprotease MEP5 | M36 |
| ARB_06651 ^s^ | protease DPPV | S09X |
| ARB_07026 ^s^ | Peptidase M14, carboxypeptidase A | M14A |
| ARB_07027 ^s^ | proteinase | M14A |
| ARB_07207 ^s^ | Peptidase S8/S53, subtilisin/kexin/sedolisin |  |
| ARB_07495 ^s^ | metalloprotease MEP1 | M43B |
| ARB_07536 ^s^ | Peptidase A1 | A01A |
| ARB_07819 ^s^ | Gamma-glutamyltranspeptidase | T03 |
| ARB_00010 | metacaspase Cas, Peptidase C14, caspase catalytic | C14B |
| ARB_01886 | aminopeptidase | M24B |
| ARB_03275 | aminopeptidase | M24X |
| ARB_04981 | aminopeptidase | S33 |
| ARB_05294 | aminopeptidase | M24B |
| ARB_06983 | aminopeptidase | M18 |
| ARB_07162 | aminopeptidase | S33 |
| ARB_07676 | aminopeptidase | M24B |
| ARB_01060 | metallopeptidase | M48A |
| ARB_01117 | metallopeptidase | M03A |
| ARB_01623 | metallopeptidase |  |
| ARB_01812 | metallopeptidase | M20A |
| ARB_02357 | metallopeptidase | M41 |
| ARB_02566 | metallopeptidase | M20A |
| ARB_03465 | metallopeptidase | M41 |
| ARB_04102 | metallopeptidase |  |
| ARB_04360 | metallopeptidase | M23B |
| ARB_04967 | metallopeptidase | M03A |
| ARB_05313 | metallopeptidase | M20A,S09X |
| ARB_06334 | metallopeptidase |  |
| ARB_07680 | metallopeptidase | M76 |
| ARB_00145 | metallopeptidaseaminopeptidase | M24A |
| ARB_00358 | metallopeptidaseaminopeptidase | M01 |
| ARB_00116 | proteinase |  |
| ARB_03789 | proteinase | M14A |
| ARB_03790 | proteinase | S08A |
| ARB_06045 | proteinase |  |
| ARB_06111 | proteinase | S08A |
| ARB_01266 | ? | S64? |
| ARB_07474 | ? | S54 |
| ARB_00494 | aminopeptidase, putative | S08A |
| ARB_03074 | asparaginase family protein | T02 |
| ARB_01619 | aspartic-type endopeptidase (CtsD), putative | A01A |
| ARB_05775 | ATP-dependent protease (CrgA), putative |  |
| ARB_02553 | autophagy cysteine endopeptidase Atg4, putative | C54 |
| ARB_07523 | beta-alanine synthase, putative | M20X |
| ARB_01185 | C6 transcription factor, putative |  |
| ARB_01292 | C6 transcription factor, putative |  |
| ARB_07403 | extracellular aspartic endopeptidase, putative | A01A |
| ARB_04095 | conserved hypothetical protein, Peptidase aspartic, active site |  |
| ARB_04097 | Proteasome, subunit alpha/beta | T01A |
| ARB_04194 | Peptidase C19, ubiquitin carboxyl-terminal hydrolase 2 | C19 |
| ARB_04355 | Peptidase S16, Lon protease | S16 |
| ARB_04528 | calpain-like protein, Peptidase C2, calpain C02A |  |
| ARB_04678 | Peptidase S8/S53, subtilisin/kexin/sedolisin | S53 |
| ARB_07953 | Peptidase S8/S53, subtilisin/kexin/sedolisin | S08B |
| ARB_08031 | rhomboid family protein, putative Peptidase S54 | S54 |
| ARB_00131 | Peptidase S8/S53, subtilisin/kexin/sedolisin | S08B |
| ARB_00199 | WLM (Wss1p-like metalloproteases), Zinc finger, RanBP2-type |  |
| ARB_00501 | Peptidase S14, ClpP | S14 |
| ARB_00555 | Peptidase S58, DmpA | S58 |
| ARB_00556 | Beta-lactamase-type transpeptidase fold | S12 |
| ARB_00417 | WW domain protein, Peptidase C14, caspase catalytic | C14B |
| ARB_00615 | Proteasome, subunit alpha/beta | T01A |
| ARB_00823 | Peptidase M16 | M16A |
| ARB_00843 | Peptidase M19, renal dipeptidase | M19 |
| ARB_00859 | MATH and UCH domain protein, putative Peptidase C19, ubiquitin carboxyl-terminal hydrolase 2 | M23B,C19 |
| ARB_00883 | conserved hypothetical protein, Peptidase C78, ubiquitin fold modifier-specific peptidase 1/ 2, Zinc finger, C2H2-like |  |
| ARB_00985 | Proteasome, subunit alpha/beta | T01A |
| ARB_00987 | Proteasome endopeptidase complex, beta subunit | T01X |
| ARB_01024 | Serine/cysteine peptidase, trypsin-like |  |
| ARB_01044 | Proteasome, subunit alpha/beta | T01A |
| ARB_01049 | Proteasome, subunit alpha/beta | T01A |
| ARB_01052 | Peptidase C19, ubiquitin carboxyl-terminal hydrolase 2 | C19 |
| ARB_01067 | Peptidase M16, | M16C |
| ARB_01098 | Peptidase M16 | M16C |
| ARB_00976 | Proteasome, subunit alpha/beta | T01A |
| ARB_01104 | Peptidase S16, Lon protease | S16 |
| ARB_01296 | Protease-associated PA, Peptidase M28 | M28X |
| ARB_01322 | Peptidase aspartic, catalytic |  |
| ARB_04820 | Peptidase S8/S53, subtilisin/kexin/sedolisin | S33 |
| ARB_04732 | Peptidase M28 | M28E |
| ARB_04970 | Peptidase T1A, proteasome beta-subunit; Proteasome, subunit alpha/beta | T01A |
| ARB_05175 | Peptidase, cysteine peptidase active site, Aminotransferase, class V/Cysteine desulfurase |  |
| ARB_05208 | Peptidase S26B, eukaryotic signal peptidase | S26B |
| ARB_05259 | Peptidase C19, ubiquitin carboxyl-terminal hydrolase 2, | C19 |
| ARB_05267 | Peptidase C19, ubiquitin carboxyl-terminal hydrolase 2 | C19 |
| ARB_05309 | Peptidase T2, asparaginase 2 | T02 |
| ARB_05331 | Proteasome, alpha-subunit, conserved site, beta-type subunit, conserved site | T01A |
| ARB_01511 | Peptidase S1/S6 | S01B |
| ARB_01652 | Peptidase, cysteine peptidase active site, BUT Transcription factor jumonji/aspartyl beta-hydroxylase |  |
| ARB_01633 | Peptidase S1/S6 | S01X |
| ARB_01820 | Peptidase M16, | M16B |
| ARB_02032 | Peptidase S10, serine carboxypeptidase | S10 |
| ARB_01985 | Peptidase C19, ubiquitin carboxyl-terminal hydrolase 2 | C19 |
| ARB_02224 | Peptidase C45, acyl-coenzyme A:6-aminopenicillanic acid acyl-transferase | C45 |
| ARB_02115 | Peptidase C12, ubiquitin carboxyl-terminal hydrolase 1 | C12 |
| ARB_02390 | Protease-associated PA, Peptidase M28 | M28X,S08A |
| ARB_02420 | Peptidase C48, SUMO/Sentrin/Ubl1 | C48 |
| ARB_02634 | Peptidase C19, ubiquitin carboxyl-terminal hydrolase 2 | C19 |
| ARB_05467 | Peptidase S8/S53, subtilisin/kexin/sedolisin, ATPase, F1 complex, OSCP/delta subunit, CCR4-Not complex component, Not1 |  |
| ARB_05486 | Peptidase, cysteine peptidase active site |  |
| ARB_05510 | Protease-associated PA, Peptidase M28 | M28X |
| ARB_05386 | Proteasome, subunit alpha/beta | T01A |
| ARB_05554 | Peptidase M28 | M28X |
| ARB_05581 | Beta-lactamase-type transpeptidase fold | S12 |
| ARB_02762 | Peptidase aspartic, catalytic | A01A |
| ARB_02878 | Peptidase C19 | C19 |
| ARB_03094 | Peptidase M19, renal dipeptidase | M19 |
| ARB_03114 | conserved hypothetical protein, Peptidase S26A, signal peptidase I |  |
| ARB_03239 | Peptidase S54, rhomboid | S54 |
| ARB_03360 | Peptidase C19, ubiquitin carboxyl-terminal hydrolase 2 | C19 |
| ARB_03448 | Peptidase, cysteine peptidase active site | M23B |
| ARB_03503 | Peptidase M14, carboxypeptidase A, Protein-L-isoaspartate(D-aspartate) O-methyltransferase |  |
| ARB_03547 | acyl-CoA:6-aminopenicillanic-acid-acyltransferase, Peptidase C45 | C45 |
| ARB_03568 | Peptidase M28 | M28E |
| ARB_05924 | conserved hypothetical protein, Peptidase S8/S53, subtilisin/kexin/sedolisin |  |
| ARB_05842 | Peptidase S26A, signal peptidase I, conserved site+ many others(ACP, lipase, transferaseà) |  |
| ARB_06218 | Peptidase S9A, oligopeptidase, WD40 repeat | S09X |
| ARB_03717 | Peptidase C12, ubiquitin carboxyl-terminal hydrolase 1 | C12 |
| ARB_03764 | Beta-lactamase-type transpeptidase fold | S12 |
| ARB_06361 | Peptidase S10, serine carboxypeptidase | S10 |
| ARB_06511 | Peptidase S9, prolyl oligopeptidase, catalytic domain | S09X |
| ARB_06590 | Peptidase S9 | S09B |
| ARB_03918 | Peptidase C14, ICE, catalytic subunit p20, active site; Protein kinase-like |  |
| ARB_06723 | Peptidase S8/S53, subtilisin/kexin/sedolisin | S08A |
| ARB_06736 | ?? Peptidase S1/S6, chymotrypsin/Hap, active site; AMP-dependent synthetase/ligase |  |
| ARB_06742 | Peptidase M24, structural domain | M24B |
| ARB_06652 | Peptidase C12, ubiquitin carboxyl-terminal hydrolase 1 | C12 |
| ARB_06863 | Peptidase C19, ubiquitin carboxyl-terminal hydrolase 2 |  |
| ARB_06882 | Peptidase M14, carboxypeptidase A |  |
| ARB_06902 | leukotriene A4 hydrolase, Peptidase M1 | M01 |
| ARB_07081 | Peptidase M16 | M16B |
| ARB_07161 | Peptidase S10, serine carboxypeptidase | S10 |
| ARB_07195 | Peptidase S41 |  |
| ARB_04336 | metalloproteinase, putative | M35 |
| ARB_06182 | methionine aminopeptidase, type I, putative | M24A |
| ARB_02691 | methionine aminopeptidase, type II, putative | M24A |
| ARB_06861 | microsomal signal peptidase subunit (gp23), putative | C19 |
| ARB_03375 | mitochondrial inner membrane protease subunit 1, putative | S26A |
| ARB_07845 | mitochondrial inner membrane protease subunit Imp2, putative | S26A |
| ARB_06755 | NACHT and WD40 domain protein | S08A,S09X |
| ARB_07937 | proteasome component Pre3, putative | T01A |
| ARB_07910 | pyroglutamyl peptidase type I, putative | C15 |
| ARB_01587 | serine carboxypeptidase, putative | S10 |
| ARB_06548 | signal peptidase complex component, putative |  |
| ARB_04944 | subtilisin-like protease SUB1 | S08A |
| ARB_06076 | subtilisin-like protease SUB7 | S08A |
| ARB_06075 | transesterase (LovD), putative | S12 |
| ARB_03874 | ubiquitin carboxyl-terminal hydrolase, putative | C12 |
| ARB_07520 | ubiquitin C-terminal hydrolase Ubp8, putative | C19 |
| ARB_04905 | ubiquitin C-terminal hydrolase, putative | C19 |
| ARB_04233 | ubiquitin-specific protease, putative | C19 |
| ARB_06307 | Ulp1 protease family protein | C48 |
| ARB_06860 | Ulp1 protease family protein | C48 |
| ARB_07733 | vacuolar carboxypeptidase Cps1, putative | M20A |
| ARB_04942 | zinc carboxypeptidase, putative | M14A |

Table S5. Identification and prediction of secretion signals of protein spots shown in Fig. 3

| **Spot/** | **Protein identification / function^1^** | **Acc. No.** | **N-terminal** | relative cluster |
| --- | --- | --- | --- | --- |
| **cluster** |  |  | **signal peptide** | **volume (%)** |
| **number** |  |  | **NN/HMM^2^** |  |
| 1 | [WSC - domain protein, galactose oxidase]* | ARB_07867 | y/n | 1.17 |
| 2 | beta-D-glucoside glucohydrolase* | ARB_05654 | y/y | 0.25 |
| 3 | glutamate carboxypeptidase, putative | ARB_02390 | y/y | 0.22 |
| 4 | alpha-1,2-mannosidase, putative subfamily* | ARB_07629 | y/y | 0.07 |
| 5 | [WSC - domain protein, haem peroxidase] | ARB_07870 | y/y | 0.02 |
| 6 | neutral/alkaline nonlysosomal ceramidase, putative | ARB_01232 | y/y | 0.02 |
| 7 | hypothetical protein | ARB_06907 | y/y | 0.04 |
| 8 | [glucoamylase]* | ARB_02327 | y/y | 0.95 |
| 9 | conserved hypothetical protein | ARB_04696 | y/y | 0.25 |
| 10 | alpha-glucosidase AgdA, putative* | ARB_02101 | y/y | 0.88 |
| 11 | feruloyl esterase, putative | ARB_07085 | y/y | 0.03 |
| 12 | feruloyl esterase, putative | ARB_02148 | y/y | 0.17 |
| 13 | carboxylesterase, putative | ARB_02369 | y/y | 0.76 |
| 14 | tripeptidyl peptidase SED3 | ARB_04101 | y/y | 0.58 |
| 15 | mannosidase MsdS* | ARB_00035 | y/y | 0.15 |
| 16 | [Cupin domain protein], oxalate decarboxylase | ARB_04859 | y/y | 0.84 |
| 17 | FAD-dependent oxygenase | ARB_05319 | yy | 1.37 |
| 18 | ribonuclease T2, putative | ARB_01347 | y/y | 0.36 |
| 19 | autophagic serine protease Alp2 = SUB7 | ARB_06076 | n.d.^3^ | 0.46 |
| 20 | exo-beta-1,3-glucanase (Exg1), putative* | ARB_04467 | y/y | 0.28 |
| 21 | Peptidase S41 family protein | ARB_02220 | y/y | 14.63 |
| 22 | 1,3-beta-glucanosyltransferase Bgt1* | ARB_02797 | y/y | 1.09 |
| 23 | conserved hypothetical protein | ARB_00595 | y/y | 0.72 |
| 24 | hypothetical protein | ARB_07590 | y/y | 0.83 |
| 25 | GPI anchored cell wall protein, putative | ARB_00344 | n.d.^3^ | 0.57 |
| 26 | BYS1 domain protein, putative | ARB_01932 | y/y | 0.15 |
| 27 | allergenic cerato-platanin Asp F13 | ARB_05304 | y/y | 0.16 |
| 28 | [phospholipase A2] | ARB_02001 | y/y | 0.15 |
| 29 | hypothetical protein | ARB_00107 | y/y | 0.37 |
| 30 | conserved hypothetical protein | ARB_04619 | y/y | 0.18 |
| 31 | metalloprotease [peptidase M43] | ARB_05317 | y/y | 0.68 |
| 32 | conserved hypothetical protein | ARB_00926 | n.d.^3^ | 0.07 |
| 33 | N,O-diacetyl muramidase, putative* | ARB_05911 | y/y | 0.16 |
| 34 | hypothetical protein | ARB_01979 | ambiguous/y | 0.33 |
| 35 | subtilisin-like protease SUB4 | ARB_01032 | y/y | 1.77 |
| 36 | subtilisin-like protease SUB3 | ARB_00701 | y/y | 3.89 |
| 37 | putative carboxypeptidase M14A | ARB_07027 | y/y | 2.22 |
| 38 | leucine aminopeptidase LAP2 | ARB_03568 | y/y | 4.17 |
| 39 | extracellular serine carboxypeptidase, putative | ARB_01345 | y/y | 2.01 |
| 40 | elastinolytic metalloproteinase Mep = MEP3 | ARB_05085 | y/y | 8.28 |
| 41 | elastinolytic metalloproteinase Mep = MEP1 | ARB_02406 | y/y | 0.52 |
| 42 | tripeptidyl peptidase SED3 | ARB_05765 | y/y | 0.65 |
| 43 | amidase family protein | ARB_02965 | y/y | 9.89 |
| 44 | serine peptidase, putative | ARB_00083 | y/y | 3.81 |
| 45 | [peptidase M14] = *T. rubrum* carboxypeptidase 2 | ARB_02407 | y/y | 0.51 |
| 46 | elastinolytic metalloproteinase Mep = MEP4 | ARB_00762 | y/y | 1.13 |
| 47 | GABA permease, putative | ARB_01417 | y/y | 2.01 |
| 48 | Class V chitinase, putative* | ARB_05392 | n/n | 0.19 |
| 49 | alkaline phosphatase Pho8 | ARB_05076 | y/y | 0.20 |
| 50 | aminopeptidase, putative | ARB_00494 | y/y | 7.79 |
| 51 | [zinc metallopeptidase] | ARB_06334 | y/y | 1.77 |
| 52 | secreted dipeptidyl peptidase DPPV | ARB_06651 | y/y | 10.33 |
| 53 | secreted dipeptidyl peptidase DPPIV | ARB_06110 | y/y | 1.74 |

*proteins putatively involved in carbohydrate metabolism and/or breakdown of carbohydrate substrates

^1^If GenBank description was not available, domain features [in brackets] were used to name the protein

^2^SignalP (http://www.cbs.dtu.dk/services/SignalP) result using neural networks (NN) and hidden Markov models (HMM) trained on eukaryotes

^3^not determined

**Table S6:** Comparison of dermatophyte secretome data of Giddey et al. [4 ] and the present study

| **Secreted Proteins on soy protein (Giddey et al. 2007)** | | | | |  | **… on keratin (present study)** | | |
| --- | --- | --- | --- | --- | --- | --- | --- | --- |
| name | Acc. No. | Cluster No. | Cluster volume | |  | Acc. No. | Cluster No. | Cluster volume |
|  |  |  | *T. rub.* | *T. viol.* |  | *A. ben.* |  |  |
| Putative beta-glucosidase | S06892 | 1 | 1.02 | 0.03 |  | ARB_05654 | 2 | 0.25 |
| Putative ceramidase | S14058 | 2 | 0.69 | 0.76 |  | ARB_01232 | 6 | 0.02 |
| Putative glucoamylase | S15010 | 3 | 0.36 | 0.04 |  | ARB_02327 | 8 | 0.95 |
| Putative Zn-metalloproteased | S12285, Q1E6A7 | 5 | 1.71 | 0.00 |  | ARB_06334 | 51 | 1.77 |
| Putative alkaline phosphatase | S00266 | 6 | 0.02 | 0.06 |  | ARB_03878 | n.a. | n.a. |
| Leucine aminopeptidase 1 (Lap1) | Q5QHG6 | 8,9 | 19.56 | 17.36 |  | ARB_00494 | 50 | 7.79 |
| Putative glucose-6-phosphate isomerase | S02324 | 11 | 0.99 | 1.21 |  | ARB_04859 | 16 | 0.84 |
| Secreted metalloprotease 4 (Mep4) | Q8NIJ4 | 12 | 0.43 | 2.51 |  | ARB_00762 | 46 | 1.13 |
| Putative exo-beta-1,3-glucanase Exg1 | S14005 | 14 | 0.47 | 0.20 |  | ARB_04467 | 20 | 0.28 |
| Subtilisin 3 (Sub3) | Q69F56 | 15,22 | 8.66 | 0.04 |  | ARB_00701 | 36 | 3.89 |
| Secreted metalloprotease 3 (Mep3) | Q6WIH8 | 16,17,21 | 7.54 | 0.31 |  | ARB_05085 | 40 | 8.28 |
| Subtilisin 7 (Sub7) ) alkaline protease 2 (Alp2) | Q8NID9 | 18 | 0.54 | 4.01 |  | ARB_06076 | 19 | 0.46 |
| Putative C-terminal peptidase S41 family | S14420 | 19 | 0.76 | 0.13 |  | ARB_02220 | 21 | 14.63 |
| Unknown function | S12835 | 20 | 0.45 | 0.00 |  | ARB_03024 | n.a. | n.a. |
| Putative GDSL lipase/acylhydrolase | S11385 | 23,30,35,36 | 1.86 | 0.00 |  | ARB_04889 | n.a. | n.a. |
| Subtilisin 4 (Sub4) | Q69F35 | 24 | 0.48 | 0.32 |  | ARB_01032 | 35 | 1.77 |
| Putative metalloprotease M43 family | S08469 | 25 | 0.00 | 0.80 |  | ARB_05317 | 31 | 0.68 |
| Subtilisin 6 (Sub6) ) Tri r 2 allergen14 | Q9UW97 | 26 | 0.00 | 0.27 |  | ARB_05307 | n.a. | n.a. |
| Putative secreted phospholipase A2 | S11917 | 29,32,34 | 1.07 | 1.13 |  | ARB_02001 | 28 | 0.15 |

**Table S7.** Differentially expressed genes of *A. benhamiae* during cocultivation with human keratinocytes. A. Top-30 up- and downregulated genes (p-value < 0.05). B. Genes of particular interest

| **A. Differentially expressed up- and downregulated genes** | | | | | |
| --- | --- | --- | --- | --- | --- |
| **Acc. No.** | **Fold** | **Name** | | **InterPro prediction** | **p-Value FDR** |
| **upregulated in presence of human keratinocytes** | | | | |  |
| ARB_02085 | 18.11 | hypothetical protein |  | | 0.011 |
| ARB_02245 | 14.27 | mitochondrial chaperone ATPase (Bcs1) |  | | 0.032 |
| ARB_02932 | 13.41 | RTA1 domain protein |  | | 0.052 |
| ARB_06358 | 10.02 | hypothetical protein |  | | 0.034 |
| ARB_04859 | 10.02 |  | oxalate decarboxylase | | 0.040 |
| ARB_00345 | 9.66 | hypothetical protein |  | | 0.044 |
| ARB_04909 | 9.66 | Cation transporter, TrkH |  | | 0.042 |
| ARB_05314 | 7.88 | Cytochrome P450 |  | | 0.055 |
| ARB_06316 | 7.49 | Rhodopsin-like GPCR superfamily |  | | 0.054 |
| ARB_01027 | 5.82 |  | MFS general substrate transporter | | 0.043 |
| **downregulated in presence of human keratinocytes** | | | | |  |
| ARB_05666 | 107.68 |  | Globin-like | | 0.004 |
| ARB_07891 | 76.73 |  | D-3-phosphoglycerate dehydrogenase | | 0.000 |
| ARB_06109 | 65.18 |  | Ubiquitin fusion degradation protein UFD1 Zinc finger, C2H2-type | | 0.000 |
| ARB_06346 | 40.1 |  | Rhodanese-like Pyridoxal phosphate-dependent enzyme, beta subunit | | 0.000 |
| ARB_04156 | 35.98 |  | Pyridoxal phosphate-dependent transferase | | 0.000 |
| ARB_06345 | 35.6 | MFS transporter |  | | 0.000 |
| ARB_03185 | 34.7 | hypothetical protein |  | | 0.013 |
| ARB_03766 | 32.01 |  | Amino_oxidase | | 0.000 |
| ARB_07951 | 23.48 | hypothetical protein |  | | 0.003 |
| ARB_05744 | 22.08 |  | methyltransferase N2227 family | | 0.002 |
| ARB_05131 | 19.56 | NRPS |  | | 0.038 |
| ARB_00334 | 18.83 |  | Zinc/iron permease | | 0.006 |
| ARB_05106 | 18.79 | N-acetylglucosamine-6-phosphate deacetylase (NagA) |  | | 0.005 |
| ARB_05525 | 18.16 | pyruvate decarboxylase |  | | 0.021 |
| ARB_04558 | 16.24 | pyridine nucleotide-disulfide oxidoreductase |  | | 0.021 |
| ARB_04052 | 15.6 |  | Cytochrome P450 | | 0.032 |
| ARB_06741 | 14.08 | hypothetical protein |  | | 0.043 |
| ARB_02786 | 14 | proline oxidase Put1 |  | | 0.007 |
| ARB_02540 | 12.81 |  | Hypothetical protein YjiA, C-terminal domain cobW | | 0.009 |
| ARB_05207 | 12.5 | hypothetical protein |  | | 0.007 |
| ARB_03674 | 12.37 | cytosolic Cu/Zn superoxide dismutase |  | | 0.013 |
| ARB_03841 | 12 | aminotransferase, classes I and II family |  | | 0.019 |
| ARB_00478 | 11.89 | hypothetical protein |  | | 0.007 |
| ARB_07370 | 11.13 | UbiD family decarboxylase |  | | 0.019 |
| ARB_06241 | 10.21 | opsin |  | | 0.040 |

| **B. Proteins with potential role in pathogenicity** | | |
| --- | --- | --- |
| **Acc. No.** | **Fold** | **Name** |
| **upregulated in presence of human keratinocytes** | | |
| ARB_04859 | 10.02 | oxalate decarboxylase* |
| ARB_06019 | 6.29 | carboxypeptidase S1 [Tr]* |
| ARB_02327 | 3.13 | zinc-binding oxidoreductase* |
| ARB_01864 | 2.59 | Ser/Thr protein phosphatase family* |
| ARB_01232 | 2.54 | neutral/alkaline nonlysosomal ceramidase* |
| ARB_06651 | 2.45 | dipeptidyl-peptidase DPPV (tri m 4 allergen) * |
| ARB_06975 | 1.58 | hydrophobin* |
| **downregulated in presence of human keratinocytes** | | |
| ARB_05131 | 19.82 | NRPS |
| ARB_05304 | 11.36 | allergenic cerato-platanin Asp F13* |
| ARB_02369 | 11.00 | carboxylesterase, putative* |
| ARB_00075 | 3.01 | cell wall protein PhiA* |
| ARB_01353 | 2.84 | beta-N-acetylhexosaminidase NagA* |
| ARB_04467 | 2.25 | exo-beta-1,3-glucanase (Exg1)* |
| ARB_02797 | 2.03 | 1,3-beta-glucanosyltransferase Bgt1* |
| ARB_01444 | 1.80 | endo-1,3-beta-glucanase Engl1* |
| ARB_01698 | 1.64 | NRPS |
| ARB_00358 | 1.54 | aminopeptidase* |

Table S8. Genes implicated in sexual reproduction and meiosis specific genes

| **Gene** |  | **Function** | ***A.benhamiae*** | ***T. verrucosum*** | ***A. fumigatus*** |
| --- | --- | --- | --- | --- | --- |
| **Mating Process** | | |  |  |  |
| Mat1-1 |  | Mating-type (alpha-box domain transcriptional activator) | GB GQ996965 | - | - |
| Mat1-2 |  | Mating-type (HMG-box transcriptional activator) | ARB_07320 | TRV_02051 | AFUA_3G06170 |
| PpgA |  | Pheromone precursor (alpha-factor like) | ARB_00978 | TRV_06463 | AFUA_6G06360 |
| PpgB |  | Pheromone precursor (for a-factor like) | ambiguous | ambiguous | ambiguous |
| KexA (Kex1) |  | Carboxypeptidase alpha-factor processing | ARB_03758 | TRV_07747 | AFUA_1G08940 |
| Sxa2 |  | Pheromone processing carboxypeptidase, putative | ARB_06414 | TRV_01049 | AFUA_2G03510 |
| KexB (Kex2) |  | Endoprotease for alpha-factor processing | ARB_00131 | TRV_06672 | AFUA_4G12970 |
| (Ste13) |  | Dipeptidyl aminopeptidase alpha-factor processing | ARB_06590 | TRV_04813 | AFUA_3G07850 |
| (Ste23) |  | Pheromone processing metallopeptidase | ARB_00823 | TRV_07483 | AFUA_5G02010 |
| Rce1 |  | CAAX prenyl protease a-factor C-terminal processing | - | - | AFUA_6G04890 |
| (Ste24) |  | CAAX prenyl protease | ARB_01060 | TRV_02831 | AFUA_4G07590 |
| Ram1 (Ste16) |  | CAAX-farnesyltransferase beta subunit | ARB_03728 | TRV_006247 | AFUA_4G10330 |
| Ram2 |  | CAAX-farnesyltransferase alpha subunit | ARB_01090 | TRV_02860 | AFUA_4G07800 |
| (Ste14) |  | CAAX-prenyl cysteine carboxymethyltransferase | ARB_04882 | TRV_06581 | AFUA_2G08420 |
| AtrD (Ste6) |  | ATP-dependent efflux pump for a-factor like pheromone | ARB_05904 | TRV_00105 | AFUA_4G08800 |
| **Mating Signalling** | | |  |  |  |
| PreB (Ste2) |  | Pheromone Receptor (for alpha-factor like pheromone) | ARB_05736 | TRV_04506 | AFUA_3G14330 |
| PreA (Ste3) |  | Pheromone Receptor (for a-factor like pheromone) | ARB_06081 | TRV_00301 | AFUA_5G07880 |
| FadA (Gpa1) |  | G-protein complex alpha subunit | ARB_06680 | TRV_04023 | AFUA_1G13140 |
| SfaD (Ste4) |  | G-protein complex beta-subunit | ARB_04406 | TRV_04825 | AFUA_5G12210 |
| GpgA (Ste18) |  | G-protein complex gamma subunit | - | - | AFUA_1G05210 |
| (Ste20) |  | Serine/threonine protein kinase MKKKK | ARB_00934 | TRV_00753 | AFUA_7G04330 |
| SteC (Ste11) |  | Serine/threonine protein kinase MKKK | ARB_07142 | TRV_04878 | AFUA_5G06420 |
| (Ste7) |  | Serine/threonine protein kinase MKK | ARB_07210 | TRV_06933 | AFUA_3G05900 |
| MpkB (Fus3) |  | Mitogen-activated protein kinase | ARB_07504 | TRV_07105 | AFUA_6G12820 |
| SteA (Ste12) |  | Transcriptional Activator. Homeodomain DNA binding | ARB_04076 | TRV_04872 | AFUA_5G06190 |
| Ste50 |  | MAPKKK cascade protein kinase regulator | ARB_03313 | TRV_02719 | AFUA_2G17130 |
| **Fruiting Body Development** | | |  |  |  |
| VeA |  | Sexual development activator | ARB_01633 | TRV_00043 | AFUA_1G12490 |
| NsdD |  | GATA-type sexual development TF | ARB_07849 | TRV_04731 | AFUA_3G13870 |
| CsnD |  | COP9 signalosome subunit | ARB_05849 | TRV_01023 | AFUA_8G05500 |
| CsnE |  | COP9 signalosome subunit | ARB_03215 | TRV_02444 | AFUA_2G16250 |
| RosA |  | C6 transcription factor | ARB_01430 | TRV_02966 | AFUA_6G07010 |
| MutA |  | Mutanase, cell wall turnover during sexual development | - | - | AFUA_2G03980 |
| StuA |  | APSES-transcription factor | ARB_07703 | TRV_07259 | AFUA_2G07900 |
| MedA |  | Transcriptional regulator Medusa | ARB_04803 | TRV_03780 | AFUA_2G13260 |
| Nc Asd-1 |  | ascus development; rhamnogalacuronase B | - | - | AFUA_4G03780 |
| AreB |  | ascus development; GATA-Zn finger transcription factor | ARB_04788 | TRV_07257 | AFUA_2G13380 |
| RadC |  | DNA repair protein RADC | ARB_01608 | TRV_03545 | AFUA_4G06970 |
| Rum1 |  | PHD transcription factor | ARB_06850 | TRV_05239 | AFUA_5G03430 |
| Mcm1 |  | MADS-box TF | ARB_02013 | TRV_05844 | AFUA_6G02110 |
| Cro1 |  | Actin cytoskeleton organization protein | ARB_03076 | TRV_06075 | AFUA_5G11600 |
| **Meiosis specific genes** | | |  |  |  |
| Spo11 |  | Creation of double strand breaks | ARB_08062 | TRV_08213 | AFUA_5G04070 |
| Hop1 |  | Meiotic chromosome pairing | ARB_07462 | TRV_03978 | AFUA_6G13050 |
| Mnd1 |  | Promotes interhomologous recombination | ARB_01462 | TRV_02970 | AFUA_4G09640 |
| Dmc1 |  | Meiotic recombination | ARB_08063 | TRV_08214 | AFUA_7G02200 |
| Rad51 |  | Repair of DNA double strand breaks | ARB_08064 | TRV_03925 | AFUA_1G10410 |
| Rad21 |  | Sister chromatid cohesion | ARB_05060 | TRV_01439 | AFUA_2G05850 |
| Msh4 |  | Meiotic recombination and Holiday junction resolution | ARB_07168 | TRV_07718 | AFUA_1G02000 |
| Msh2 |  | Meiotic recombination and Holiday junction resolution | ARB_01205 | TRV_05311 | AFUA_3G09850 |
| Msh6 |  | Meiotic recombination and Holiday junction resolution | ARB_00228 | TRV_05220 | AFUA_4G08300 |
| Msh5 |  | Meiotic recombination and Holiday junction resolution | ARB_02806 | TRV_01907 | AFUA_1G11170 |

**Table S9**. Numbers of reads obtained in the transcriptome analysis of infection and control samples.

|  | **Infection**  **(with keratinocytes)** | | **Control**  **(without keratinocytes)** | |
| --- | --- | --- | --- | --- |
| Replicates | R1 | R2 | R1 | R2 |
| **Total number of reads** | 115,662 | 348,833 | 166,385 | 410,260 |
| **Mapped to genome** | 85,681 | 323,693 | 122,030 | 383,124 |
| **In gene models** | 67,474 | 269,798 | 97,515 | 324,738 |
| **NCBI accession number** | SRR070551 | SRR070552 | SRR070553 | SRR070554 |
