# Supplementary material for: Comparative and functional genomics provide insights into the pathogenicity of dermatophytic fungi
Source: Genome Biol. 2011 Jan 19;12(1):R7. doi: 10.1186/gb-2011-12-1-r7 (PMC3091305; doi:10.1186/gb-2011-12-1-r7)
Supplement: Additional file 7 — Table S4: secreted proteases in A. benhamiae, T. verrucosum, Aspergillus fumigatus and Coccidioides spp. [file gb-2011-12-1-r7-S7.DOC]

**Additional Table S4.** Secreted proteases in *A. benhamiae*, *T. verrucosum*, *Aspergillus fumigatus* and *Coccidioides* spp.

| Family/ Sub-family | Protein description | *A. fumigatus*^1^ | *A. benhamiae*^2^ | *T. verrucosum*^3^ | | *C. immitis*^4^ | *C. posadasii*^5^ |
| --- | --- | --- | --- | --- | --- | --- | --- |
|  |  |  |  |  |  | |  |
| A1 | Aspartic endopeptidase PEP2 (Vacuolar protease A) | AFUA_3G11400  O42630 | ARB_02919  D4B385 | TRV_05606  D4DEN7 | CIMG_03687 | | CPC735_005950  C5P9L1 |
| A1 | Aspartic endopeptidase PEP1 (Aspergillopepsin I) | AFUA_5G13300  P41748 | ARB_05728  D4ANC3 | TRV_03007  D4D7C5 | CIMG_00311 | | CPC735_058790  **C5PEI9** |
| A1 | Aspartic-type endopeptidase ctsD | AFUA_4G07040  Q4WNV0 | ARB_01619  D4AZK1 | TRV_03534  D4D8U6 | CIMG_04143 | | CPC735_002710 **C5P8P0** |
| A1 | Putative aspergillopepsin A-like aspartic endopeptidase | AFUA_2G15950  Q4WZS3 | ARB_07403  D4AT39 | TRV_06366  D4DGR1 | - | | - |
| A1 | Probable aspartic-type endopeptidase (opsB) | AFUA_6G05350  Q4WDN4 | ARB_04170  D4AIS3 | TRV_06035  D4DFT3 | CIMG_02633 | | CPC735_037240  **C5P2B5** |
| A1 | Aspartic-type endopeptidase, putative | - | ARB_04018  D4AIC4 | TRV_05382  D4DE18 | - | | - |
| A1 | Aspartic-type endopeptidase, putative | AFUA_3G01220  Q4WFS2 | - | - | - | | - |
| A1 | Aspartic endopeptidase, putative | AFUA_6G03260 Q4WD26 | - | - | - | | - |
| G1 | Aspergillopepsin, putative | AFUA_3G02970  Q4WF96 | - | - | - | | - |
| G1 | Aspergillopepsin, putative | AFUA_7G01200  **Q4WAG2** | - | - | - | | - |
| M12 | ADAM family of metalloprotease ADM-A | AFUA_6G14420 [Q58I96](http://www.uniprot.org/uniprot/Q58I96) | - | - | - | | - |
| M12 | ADAM family of metalloprotease ADM-*B* | AFUA_4G11150 Q4WQ08 | ARB_02289 D4B1G0 | TRV_04965 D4DCV9 | CIMG_01504 | | CPC735_049420  C5PGB9 |
| M14 | Metallocarboxypeptidase, putative | AFUA_2G08790  Q4X1U0 | ARB_04942  D4AKU7 | TRV_07128  D4DIW7 | CIMG_00817 | | CPC735_054920  C5PHW9 |
| M14 | Metallocarboxypeptidase A  (MCPA) | - | ARB_07026 / ARB_07027  D4AS12 | TRV_07931  D4DL57 | CIMG_07026 | | CPC735_050310  C5PGK8 |
| M14 | Metallocarboxypeptidase A-like protein (MCPAL) | - | ARB_03789  D4B5N0 | TRV_02598  D4D675 | - | | CPC735_033800  C5P5Q4 |
| M14 | Carboxypeptidase 2  Metallocarboxypeptidase B (MCPB) | - | ARB_02407  D4B1S6 | TRV_02159  D4D4Z1 | - | | - |
| M19 | Dipeptidase 1 | - | ARB_02715  D4B2N2 | TRV_05564  D4DEJ7 | CIMG_09127 | | CPC735_015490  C5PCZ0 |
| M19 | Dipeptidase 1-like | - | - | - | CIMG_08988 | | CPC735_014430  C5PCN6 |
| M20A | Probable peptidase | AFUA_6G06800  Q4WNC9 | ARB_01041  D4AXX2 | TRV_02791  D4D6R4 | CIMG_07326 | | CPC735_001200  C5PE42 |
| M20A | Vacuolar carboxypeptidase Cps1 | AFUA_3G07040  Q4WWU0 | - | - | - | | - |
| M20D | Amidohydrolase, putative | AFUA_1G11250  Q6MZ08 | - | - | CIMG_06892 | | CPC735_032870  **C5P5G1** |
| M24 | Methionine aminopeptidase | AFUA_4G06930  Q4WNT9 | ARB_02691  D4B2G3 | TRV_05431  D4DE65 | CIMG_04126 | | CPC735_002850  **C5P8Q4** |
| M24 | Peptidase D | AFUA_1G14920  Q4WRV9 | ARB_01886  D4B0B2 | TRV_02643  D4D6B8 | CIMG_03282 | | CPC735_009100  C5PAH2 |
| M28A | Aminopeptidase Y (Leucine aminopeptidase 2) | AFUA_3G00650  Q4WFX9 | ARB_00494  D4AWC9 | TRV_01590  D4D3D1 | CIMG_07629 | | CPC735_043150  **C5PB87** |
| M28A | Aminopeptidase, putative | AFUA_2G00220  Q4WIY5 | - | - | - | | - |
| M28E | Leucine aminopeptidase 1 | AFUA_4G04210  Q4W9P4 | ARB_03568  D4B528 | TRV_06599  D4DHE3 | - | | - |
| M28E | Leucine aminopeptidase like | - | - | - | CIMG_06741 | | CPC735_031780  **C5P552** |
| M28E | Probable leucine aminopeptidase | - | ARB_01443  D4AZ23 | TRV_05286  D4DDS4 | CIMG_08650 | | CPC735_062540  C5P3W3 |
| M28E | Probable leucine aminopeptidase | - | ARB_03492  D4B4V2 | TRV_05750  D4DF09 | - | | - |
| M28E | Probable leucine aminopeptidase | - | ARB_00576  D4AWL0 | TRV_02148  D4D4Y0 | CIMG_04518 | | CPC735_072610  **C5P1M1** |
| M28 | Peptidase family M28 protein | - | ARB_04732  D4AM42 | TRV_03476  D4D8N9 | - | | - |
| M28 | Putative glutamate carboxypeptidase | - | ARB_02390  D4B1R0 | TRV_02192  D4D523 | CIMG_01443 | | CPC735_049960  C5PGH3 |
| M28 | Glutaminyl-peptide cyclotransferase | AFUA_4G08280  Q4WP74 | ARB_00230  D4AVL6 | TRV_05222  D4DDL1 | CIMG_08829 | | CPC735_063510  **C5P460** |
| M35 | Probable neutral protease 2 homolog | AFUA_4G13750  Q4WQR6 | ARB_00849  D4AXC1 | TRV_07507/  TRV_07508  DADJW4/  D4DJW5 | - | | - |
| M35 | Probable neutral protease 2 homolog | - | ARB_03949  D4B639 | TRV_02539  D4D616 | - | | - |
| M35 | Probable neutral protease 2 homolog | - | ARB_04336  D4AJ87 | TRV_03208  D4D7X4 | - | | - |
| M35 | Probable neutral protease 2 homolog | - | - | - | CIMG_10101 | | CPC735_012270  C5NZL6 |
| M35 | Probable neutral protease 2 homolog | - | - | - | CIMG_05736 | | CPC735_019360  C5PE18 |
| M35 | Probable neutral protease 2 homolog | - | - | - | CIMG_11800 | | CPC735_031330  C5P507 |
| M35 | Probable neutral protease 2 homolog | - | - | - | CIMG_00508 | | CPC735_057220  C5PIJ9 |
| M35 | Probable neutral protease 2 homolog | - | - | - | CIMG_03010 | | CPC735_000180  C5NZY5 |
| M35 | Probable neutral protease 2 homolog (Deuterolysin B) | - | ARB_05817  D4ANL2 | TRV_05367  D4DE03 | - | | - |
| M35 | Probable neutral protease 2 homolog (Deuterolysin A) | AFUA_4G02700  Q4WA45 | ARB_04769  D4AM79 | TRV_06370  D4DGR5 | - | | - |
| M35 | Probable neutral protease 2 homolog (Deuterolysin A-like) | - | - | - | CIMG_07349 | | CPC735_001400  C5PE62 |
| M35 | Probable neutral protease 2 homolog (Deuterolysin A-like) | - | - | - | CIMG_08613 | | CPC735_062250  C5P3T4 |
| M36 | Extracellular metalloproteinase 1 (Fungalysin MEP1) | - | ARB_02406  D4B1S5 | TRV_02160  D4D4Z2 | CIMG_06073 | | CPC735_026430  C5P7A7 |
| M36 | Extracellular metalloproteinase Fungalysin MEP1-like | - | - | - | CIMG_10191 | | CPC735_012920  C5NZT1 |
| M36 | Extracellular metalloproteinase 2  (Fungalysin MEP2) | - | ARB_01382  D4AYW3 | TRV_01237  D4D2D2 | - | | - |
| M36 | Extracellular metalloproteinase 3  (Fungalysin MEP3) | - | ARB_05085  D4AL88 | TRV_06691  D4DHN5 | - | | - |
| M36 | Extracellular metalloproteinase 4  (Fungalysin MEP4) | - | ARB_00762  D4AX35 | TRV_00081  D4CZ44 | - | | - |
| M36 | Extracellular metalloproteinase 5  (Fungalysin MEP5) | AFUA_8G07080  [P46075](http://www.uniprot.org/uniprot/P46075) | ARB_06472  D4AQG5 | TRV_07092  D4DIT1 | - | | - |
| M43 | Probable metalloproteinase | AFUA_1G07730  Q4WJ01 | ARB_05317  D4ALW9 | TRV_06892  D4DI84 | CIMG_08674 | | CPC735_062670  **C5P3X6** |
| M43 | Probable metalloproteinase | - | ARB_07495  D4ATD1 | TRV_07111  D4DIV0 | - | | - |
| S8A | Subtilisin-like protease 1 (SUB1) | - | ARB_04944  D4AKU9 | TRV_07130  D4DIW9 | - | | - |
| S8A | Subtilisin-like protease 2 (SUB2) | AFUA_4G11800  P28296 | ARB_01495  D4AZ75 | TRV_08059  (fragment)  D4DLI5 | - | | - |
| S8A | SUB2-like protease | - | - | - | CIMG_09744 | | CPC735_023170  C5P6D1 |
| S8A | SUB2-like protease | - | - | - | CIMG_03989 | | CPC735_003880  C5P906 |
| S8A | SUB2-like protease | - | - | - | CIMG_10288 | | CPC735_013710  C5NZ70 |
| S8A | Subtilisin-like protease 3 (SUB3) | - | ARB_00701  D4AWY5 | TRV_07976  D4DLA2 | - | | - |
| S8A | Subtilisin-like protease 4 (SUB4) | - | ARB_01032  D4AXW3 | TRV_02781  D4D6Q4 | CIMG_05557 | | CPC735_066880  C5PCB1 |
| S8A | SUB3/4-like protease | - | - | - | CIMG_10193 | | CPC735_012930  C5NZT2 |
| S8A | SUB3/4-like protease | - | - | - | CIMG_09616 | | CPC735_024010  C5P6L5 |
| S8A | Subtilisin-like protease 5 (SUB5) | - | ARB_02223  D4B194 | TRV_00550  D4D0F5 | - | | - |
| S8A | Subtilisin-like protease 6 (SUB6) | - | ARB_05307  D4ALV9 | TRV_02343  D4D5H3 | - | | - |
| S8A | Subtilisin-like protease 7 (SUB7) | - | ARB_06076  D4APA9 | TRV_00296  D4CZQ4 | CIMG_01394 | | CPC735_050320  C5PGK9 |
| S8A | SUB6/7-like protease | - | - | - | CIMG_09106 | | CPC735_015300  C5PCX1 |
| S8A | SUB6/7-like protease | - | - | - | CIMG_10287 | | CPC735_013700  C5NZ69 |
| S8A | SUB6/7-like protease | - | - | - | CIMG_03747 | | CPC735_005570  C5P9H3 |
|  | Subtilisin-like protease 8 (SUB8) | AFUA_5G09210  P87184 | ARB_00777  D4AX50 | TRV_07778  D4DKQ4 | CIMG_13072 | | CPC735_031240  **C5P4Z8** |
| S8A | Subtilisin-like protease 9 (SUB9) | - | ARB_03790  D4B5N1 | TRV_02597  D4D674 | CIMG_07023 | | CPC735_033790  C5P5Q3 |
| S8A | Subtilisin-like protease 10 (SUB10) | - | ARB_06467  D4AQG0 | TRV_07087  D4DIS6 | - | | - |
| S8A | Subtilisin-like protease 11 (SUB11) | - | ARB_06111  D4APE3 | TRV_00097  D4CZ60 | CIMG_02881 | | CPC735_035780  C5P1W9 |
| S8A | SUB11- like protease | - | - | - | CIMG_01750 | | CPC735_047380  C5PFR5 |
| S8A | Subtilisin-like protease 12 (SUB12) | - | ARB_06416  D4AQA9 | TRV_01047  D4D1U5 | - | | - |
| S8 | Putative serine protease | - | - | - | CIMG_09348 | | CPC735_017270  C5PDG4 |
| S8B | Pheromone processing endoprotease (KexB) | AFUA_4G12970  Q4WQI8 | ARB_00131  D4AVC2 | TRV_06672  D4DHL6 | CIMG_00625 | | CPC735_056300  **C5PIA7** |
| S8B | Pheromone processing endoprotease (KexB-like) | - | ARB_07953  D4AUN6 | TRV_04084  D4DAD7 | - | | - |
| S8 | Alkaline serine protease (PR1)/allergen F18-like | AFUA_7G04930  Q4WGG2 | - | - | - | | - |
| S9B | Dipeptidyl peptidase 4 | AFUA_4G09320  Q4WPH9 | ARB_06110  D4APE2 | TRV_00096  D4CZ59 | CIMG_04442 | | CPC735_073130  **C5P1S3** |
| S9; not assigned to subfamily | Dipeptidyl-peptidase 5 | AFUA_2G09030  **P0C959** | ARB_06651  D4ARB1 | TRV_02418  D4D5P5 | CIMG_06782 | | CPC735_032100  **C5P584** |
| # S9; no subfamily | Oligopeptidase family protein | AFUA_8G04730  Q4WCE3 | ARB_06511  D4AQK4 | TRV_06200  D4DG98 | CIMG_10883 | | CPC735_038510  **C5P2P9** |
| S10 | Carboxypeptidase S1 homolog A | AFUA_5G07330  Q5VJK9 | ARB_04046  D4AIF1 | TRV_05031  D4DD24 | CIMG_11666 | | CPC735_026300  **C5P794** |
| S10 | Carboxypeptidase S1 homolog B | AFUA_8G04120  Q4WCK3 | ARB_06019  D4AP52 | TRV_01182/  TRV_01183  D4D280/  D4D281 | CIMG_04416 | | CPC735_073320  C5P1U2 |
| S10 | Carboxypeptidase S1 homolog | AFUA_1G00420  Q4WL26 | - | - | CIMG_13006 | | - |
| S10 | Carboxypeptidase S1 homolog | - | ARB_06361  D4AQ54 | TRV_07425  D4DJQ7 | CIMG_10499 | | CPC735_059650  C5PF15 |
| S10 | Carboxypeptidase S1 homolog | - | ARB_04807  D4AKG4 | TRV_03784  D4D9J1 | CIMG_05080 | | CPC735_068230  C5P0D3 |
| S10 | Carboxypeptidase Y homolog | AFUA_6G13540  Q5VJG9 | ARB_01491  D4AZ71 | TRV_08055  D4DLI1 | CIMG_02815 | | CPC735_036210  **C5P212** |
| S10 | Carboxypeptidase Y homolog | AFUA_5G14610  Q4WW68 | ARB_05721  D4ANB6 | TRV_02994  D4D7B2 | - | | - |
| S10 | Carboxypeptidase Y homolog | - | ARB_07161  D4ASE6 | TRV_07724/  TRV_07725  D4DKK2/  D4DKK3 | CIMG_09968 | | CPC735_011420  C5NZD1 |
| S10 | Carboxypeptidase Y homolog | AFUA_5G01200  Q4WDZ3 | - | - | - | | - |
| S10 | Carboxypeptidase Y homolog | AFUA_3G12210  Q4WY91 | - | - | - | | - |
| S10 | Carboxypeptidase cpdS homolog | AFUA_6G00310  Q4W8Y5 | ARB_02032  D4B0Q6 | TRV_05861  D4DFB5 | CIMG_02284 | | CPC735_039410  **C5P2Y9** |
| S10 | Carboxypeptidase cpdS homolog | AFUA_2G03510  Q5VJG7 | ARB_06414  D4AQA7 | TRV_01049  D4D1U7 | - | | - |
| S10 | Carboxypeptidase cpdS homolog | AFUA_4G07270  Q4WNX3 | ARB_01587  D4AZG9 | TRV_03565  D4D8X7 | CIMG_04074 | | CPC735_003250  **C5P8U4** |
| S10 | Pheromone processing carboxypeptidase Kex1 | AFUA_1G08940  Q4WTK9 | ARB_03758  D4B5L8 | TRV_07747  D4DKM5 | CIMG_07181 | | CPC735_035110  **C5P635** |
| S26 | Microsomal signal peptidase subunit | AFUA_3G12840  Q4WYF4 | ARB_05208  D4ALL0 | TRV_02351  D4D5I1 | CIMG_03464 | | CPC735_007680  **C5PA33** |
| S28 | Serine peptidase, family S28, putative | AFUA_2G01250  Q4WIN2 | - | - | - | | - |
| S28 | Serine peptidase, family S28, putative | AFUA_4G03790  Q4W9T5 | ARB_00083  D4AV74 | TRV_00921  D4D1H1 | CIMG_09541 | | CPC735_018740  **C5PDV9** |
| S28 | Serine peptidase, family S28, putative | AFUA_2G17330  Q4WZD5 | ARB_01345  D4AYS6 | TRV_01922  D4D4A7 | CIMG_04983 | | CPC735_069010  C5P0L1 |
| S41 | Peptidase S41 family protein | - | ARB_02220  D4B191 | TRV_00470  D4D076 | - | | - |
| S41 | Peptidase S41 family protein | - | ARB_02997  D4B3G0 | TRV_01713  D4D3Q3 | CIMG_02067 | | CPC735_045090  C5PEI1 |
| S41 | Peptidase S41 family protein | - | ARB_07195  D4ASI0 | TRV_03097  D4D7L4 | - | | - |
| S41 | Peptidase S41 family protein | AFUA_1G17400  Q4WR63 | - | - | - | | - |
| S53 | Tripeptidyl-peptidase SED1  (Sedolisin-A) | AFUA_6G10250  Q70DX9 | - | - | - | | - |
| S53 | Tripeptidyl-peptidase SED2  (Sedolisin-B) | AFUA_4G03490  Q70J59 | ARB_05765  D4ANG0 | TRV_04476  D4DBH6 | - | | - |
| S53 | Tripeptidyl-peptidase SED2-like  (Sedolisin-B-like) | - | - | - | CIMG_00167 | | CPC735_059790  C5PF29 |
| S53 | Tripeptidyl-peptidase SED2-like  (Sedolisin-B-like) | - | - | - | CIMG_02188 | | CPC735_038590  C5P2Q7 |
| S53 | Tripeptidyl-peptidase SED3  (Sedolisin-C) | AFUA_3G08930  Q70GH4 | ARB_04677  D4AK75 | TRV_03120  D4D7N6 | - | | - |
| ? | Tripeptidyl-peptidase SED4  (Sedolisin-D) | AFUA_4G14000  Q4WQU0 | ARB_04101  D4AIK6 | TRV_03885  D4D9U2 | CIMG_03887 | | CPC735_004570  C5P974 |
| S53 | Tripeptidyl-peptidase SED5  (Sedolisin-E) | AFUA_7G06220  Q4WGU1 | - | - | - | | - |
| S53 | Alpha/beta fold family hydrolase, putative | AFUA_1G11400  Q6MYZ3 | - | - | - | | - |

^1^ *A. fumigatus* complete genome of strain [Af293 / CBS 101355 / FGSC A1100](http://www.uniprot.org/uniprot/?query=organism:5085+strain:"Af293+/+CBS+101355+/+FGSC+A1100") (Nierman *et al*., 2005)

^2^ *A. benhamiae* complete genome of strain CBS 113480 (Burmester *et al*., Submitted to GenBank/EMBL in 2009)

^3^ *T. verrucosum* complete genome of strain HKI 0517 (Burmester *et al*., Submitted to GenBank/EMBL in, 2009)

^4^ *C. immitis* complete genome of strain RS

^5^ *C. posadasii* complete genome of strain C735 (Sharpton *et al*., 2009)

^1,2,3,5^ Proteins are from UniProtKB database (http://www.uniprot.org/). UniProtKB accession numbers are indicated below the gene name. Closest homologs were identified by Blast search using the *A. fumigatus* and/or *M. canis* proteins as query.

# ^4^ Proteins are from the Broad Institute Coccidioides Group Database (http://www.broadinstitute.org/annotation/genome/coccidioides_group/MultiHome.html). Closest homologs were identified by Blast search using the A. fumigatus and/or M. canis proteins as query.

Colour code:

Yellow - dermatophyte-specific proteases (found in dermatophytes, but not in *Coccidioides* and *Aspergillus*);

Blue –*Coccidioides*-specific;

Purple –*Aspergillus*-specific;

Green – found in *Aspergilli* and dermatophytes, but not in *Coccidioides*;

Pink - found in *Coccidioides* and dermatophytes, but not in *Aspergilli*;

Grey - found in *Aspergilli* and *Coccidioides*, but not in dermatophytes;

White – found in all species.
