# Supplementary material for: Comparative and functional genomics provide insights into the pathogenicity of dermatophytic fungi
Source: Genome Biol. 2011 Jan 19;12(1):R7. doi: 10.1186/gb-2011-12-1-r7 (PMC3091305; doi:10.1186/gb-2011-12-1-r7)
Supplement: Additional file 8 — Phylogenetic trees of secreted proteases. The file contains the phylogenies of the A. benhamiae, T. verrucosum, and Coccidioides secreted proteases of the most distinguishing families S8, M35, and M36 (Figure S3.1, S3.2, and S3.3, respectively). [file gb-2011-12-1-r7-S8.DOC]

Additional file 7 - Phylogentic trees of the *A. benhamiae*, *T. verrucosum*, and *Coccidioides* spp. secreted proteases.

1000

1000

1000

1000

1000

1000

1000

1000

1000

1000

1000

1000

1000

1000

1000

1000

1000

1000

1000

1000

1000

1000

1000

1000

1000

1000

1000

840

1000

840

1000

1000

880

585

1000

758

1000

1000

770

770

1000

960

860

1000

780

920

CP 033790

CIMG 07023

ARB 03790

TRV 02597

CP 012930

CIMG 10193

CP 024010

CIMG 09616

CP 066880

CIMG 05557

ARB 01032

TRV 02781

ARB 00701

TRV 07976

ARB 06111

TRV 00097

CP 035780

CIMG 02881

ARB 06416

TRV 01047

CP 047380

CIMG 01750

ARB 04944

TRV 07130

ARB 02223

TRV 00550

CP 005570

CIMG 03747

CP 015300

CIMG 09106

CP 013700

CIMG 10287

CP 050320

CIMG 01394

ARB 06076

TRV 00296

ARB 05307

TRV 02343

ARB 06467

TRV 07087

ARB 00777

TRV 07778

CP 031240

CIMG 13072

CP 023170

CIMG 09744

CP 003880

CIMG 03989

ARB 01495

TRV 08059

CP 013710

CIMG 10288

990

1000

1000

1000

0.1

**Fig. S3.1.** S8 family in *A. benhamiae*, *T. verrucosum*, and *Coccidioides* spp. Red color corresponds to dermatophytes, yellow – to the *Coccidioides*.

**Fig. S3.2.** M35 family in *A. benhamiae*, *T. verrucosum* (red color both), *Coccidioides* spp. (yellow), and *A. fumigatus* (blue).

CPC735 012920

CIMG 10191T0

1000

Afu8g07080

875

CPC735 026430

CIMG 06073T0

1000

ARB 02406

TRV 02160

1000

ARB 06472

TRV 07092

1000

1000

965

1000

ARB 00762

TRV 00081

1000

705

ARB 01382

TRV 01237

1000

ARB 05085

TRV 06691

1000

TRICHOTOMY

0.1

**Fig. S3.3.** M36 family in *A. benhamiae*, *T. verrucosum* (both – red color), *Coccidioides* spp. (yellow), and *A. fumigatus* (blue).
