# Supplementary material for: Comparative and functional genomics provide insights into the pathogenicity of dermatophytic fungi
Source: Genome Biol. 2011 Jan 19;12(1):R7. doi: 10.1186/gb-2011-12-1-r7 (PMC3091305; doi:10.1186/gb-2011-12-1-r7)
Supplement: Additional file 9 — Figure S4: Northern Blot analysis. [file gb-2011-12-1-r7-S9.DOC]

| Gene | ARB_04092 Actin  control | |  | ARB_00831 GAP-DH  control | |  | ARB_07891 D-3-phosphoglycerate dehydrogenase | |  | ARB_04156 Pyridoxal phosphate-dependent transferase | |
| --- | --- | --- | --- | --- | --- | --- | --- | --- | --- | --- | --- |
| Gel | 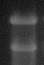 | 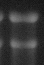 |  | 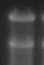 | 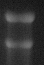 |  | 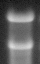 | 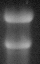 |  | 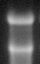 | 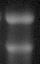 |
| ID | control | sample |  | control | sample |  | control | sample |  | control | sample |
| Blot | 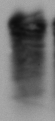 | 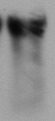 |  | 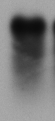 | 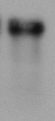 |  | 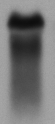 | 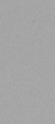 |  | 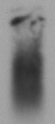 | 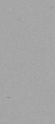 |
| Intensity | 8093 | 6048 |  | 7269 | 4591 |  | 7947 | 68 |  | 5146 | 52 |
| Fold change | 0.747 | |  | 0.632 | |  | 0.009 (x116.9) | |  | 0.010 (x98.69) | |
| comment | not diff. regulated | |  | not diff. regulated | |  | down - regulated | |  | down - regulated | |
|  |  | |  |  | |  |  | |  |  | |
| Gene | ARB_06975 Hydrophobin Hyp1 | |  | ARB_01650 Carbohydrate/ purine kinase, PfkB | |  | ARB_01027 MFS general substrate transporter | |  | ARB_04859 Oxalate decarboxylase | |
| Gel | 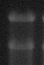 | 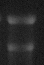 |  | 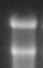 | 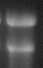 |  | 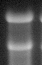 | 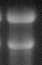 |  | 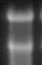 | 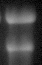 |
| ID | control | sample |  | control | sample |  | control | sample |  | control | sample |
| Blot | 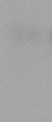 | 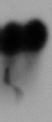 |  | 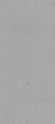 | 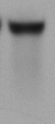 |  | 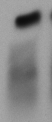 | 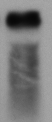 |  | 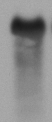 | 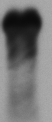 |
| Intensity | 237 | 9496 |  | 30 | 4180 |  | 4095 | 10764 |  | 3365 | 12084 |
| Fold change | 40.07 | |  | 139.3 | |  | 2.629 | |  | 3.591 | |
| comment | up - regulated | |  | up - regulated | |  | up - regulated | |  | up - regulated | |

**Fig. S4: Northern Blot analysis.** Intensities are normalized arbitrary units, calculated based on mRNA optical density on gel and blot signal strength.
