# Supplementary material for: Comparative and functional genomics provide insights into the pathogenicity of dermatophytic fungi
Source: Genome Biol. 2011 Jan 19;12(1):R7. doi: 10.1186/gb-2011-12-1-r7 (PMC3091305; doi:10.1186/gb-2011-12-1-r7)
Supplement: Additional file 10 — Phylogenetic trees of A. benhamiae, T. verrucosum, and Coccidioides immitis PKSs and NRPSs. The file contains phylogenetic trees built for NRPSs (Figure S5.1) and PKSs (Figure S5.2), comparing the corresponding genes sets of the three species. [file gb-2011-12-1-r7-S10.DOC]

### Fig. S5.1. NRPS phylogenic tree built for *A. benhamiae*, *T. verrucosum*, and *Coccidioides immitis*. *C. immitis* NRPSs are marked with blue

ARB 03095 T-C-A-T-C

TRV 06056 T-C-A

1000

TRV 01781 T-C-A-T-C

1000

ARB 07862 A-T-C-A-T-C-T

TRV 04720 A-T-C-A-T-C-T

1000

1000

ARB 02226 A-T-C-A-T-C-A-T-C

TRV 00553 A-T-C-A-T-C-A-T-C

1000

1000

ARB 07686 A-T-C-A-T-C-T-C-A-T-C-T-C-T-C

TRV 05452 A-T-C-A-T-C-T-C-A-T-C-T-C-T-C

1000

CIMG 00941 A-T-C-A-T-C-T-C-A-T-C-T-C-T

1000

1000

ARB 00195 A-T-C-T-C

TRV 05651 A-T-C-T-C

1000

CIMG 07298 A-T-C-T

1000

CIMG 01429 A-T-C-T

790

980

TRV 01776 A-T-C-A-T

ARB 7850 A-T-C

1000

570

ARB 04984 A-T-C-A-T-C

TRV 06313 A-T-C-A-T-C

1000

CIMG 01861 A-T-C-A-T

1000

820

ARB 01698 C-A-T-C-A-T-C-A-T-C-A-T-C-A-T-C-T-C-T

TRV 01735 A-T-C-A-T-C-A-T-C-A-T-C-A-T-C-T-C-T

1000

CIMG 09750 A-T-C-A-T-C-A-T-C-A-T-C-A-T-C-T-C-T

1000

ARB 02750 A-T-C-A-T-C-A-T-C-A-T-C-A-T-C-T

TRV 06186+85 A-T-C-A-T-C-A-T-C-A-T-C-A-T-C-T

1000

1000

930

ARB 02569+70 ATC

TRV 05508+07 ATC

1000

TRV 05681 A-T-C

720

ARB 06786 A-T-C

1000

ARB 05579 T-C-A-T-C-A-T

TRV 06828 T-C-A-T-C-A-T

1000

940

ARB 03768 A-C-A-T-C-A-T

TRV 07570 A-C-A-T-C-A-T

1000

1000

ARB 02149 C-A-T-C-A-T-C-A-T-C-A-T-C

1000

ARB 05131 A-T-C-A-T-C-A-T

TRV 07837 A-T-C-A-T-C-A-T

1000

930

0.1

**Fig. S5.2.** PKS phylogenic tree built for *A. benhamiae*, *T. verrucosum*, and *Coccidioides immitis*. *C. immitis* NRPSs are blue. 2 bacterial PKSs are used as an outgroup. Reducing and non-reducing PKSs are distinguished by the blue and yellow background, respectively.

ARB 07933 KS-AT-ME-ER-KR-ACP

1000

1000

1000

1000

CIMG 13632 KS-AT-ER-KR-ACP

1000

1000

CIMG 03014 KS-AT-DH-ER-KR-ACP

CIMG 05569 KS-AT-DH-ER-KR-ACP

980

**ARB 07534 KS-AT-DH-ER-KR-ACP-C-A-T**

**TRV 00508 KS-AT-DH-ER-KR-ACP-C-A-T**

1000

1000

825

ARB 05333 KS-AT-DH-ME-ER-KR-ACP

TRV 06912 KS-AT-DH-ME-ER-KR-ACP

1000

CIMG 02398 KS-AT-DH-KR-ACP

1000

1000

**ARB 02973 KS-AT-ME-KR-ACP-C-A-T**

**TRV 03721 KS-AT-ME-KR-ACP-C-A-T**

1000

1000

1000

**CIMG 06629 KS-AT-DH-ME-KR-ACP-C-A-T**

1000

1000

TRV 02519 KS-AT-ACP-ME

1000

CIMG 13102 KS-AT-ACP ME

1000

CIMG 05571 KS-AT-ACP

1000

CIMG 04689 KS AT-ACP-ME

CIMG 03162 KS-AT-ACP

1000

1000

ARB 07994 KS-AT-ACP-ACP-TE

TRV 04611 KS-AT-ACP-ACP-TE

1000

CIMG 08569 KS-AT-ACP-ACP-TE

1000

ARB 00538 KS-AT-ACP

TRV 00386 KS-AT-ACP

1000

1000

990

CIMG 08564 AT-KS-ACP-TE

990

980

ARB 05854 KS-AT-KR-ACP

TRV 06867 KS-AT-KR-ACP

1000

1000

ARB 07844 **A-T-KS-AT-KR-ACP**

TRV 05146 **A-T-KS-AT-KR-ACP**

1000

1000

Streptomyces coelicolor A3 2 NP 630373 type I PKS

Streptomyces avermitilis MA-4680 NP 824073 PKS

1000

0.1

ARB 01525 KS-AT-ME-ER-KR-ACP

TRV 04104 KS-AT-ME-ER-KR-ACP

ARB 06393 KS-AT-ME-ER-KR-ACP

TRV 01071 KS-AT-ME-ER-KR-ACP

TRV 04236 KS-AT-ME-ER-KR-ACP

ARB 07966 KS-AT-ME-KR-ACP

TRV 04285 KS-AT-ME-KR-ACP

ARB 03291 KS-AT-ACP-ME

PKS-NRPS hybrids

partially reducing

(6-MSAS)

PKS-NRPS hybrid

Bacterial outgroup

**Reducing PKSs**

**Non-reducing PKSs**
